# Towards a prebiotic chemoton – nucleotide precursor synthesis driven by the autocatalytic formose reaction†

**DOI:** 10.1039/d3sc03185c

**Published:** 2023-08-29

**Authors:** Quoc Phuong Tran, Ruiqin Yi, Albert C. Fahrenbach

**Affiliations:** a School of Chemistry, University of New South Wales Sydney NSW 2052 Australia a.fahrenbach@unsw.edu.au; b Australian Centre for Astrobiology, University of New South Wales Sydney NSW 2052 Australia; c UNSW RNA Institute, University of New South Wales Sydney NSW 2052 Australia; d Earth-Life Science Institute, Tokyo Institute of Technology Tokyo 152-8550 Japan

## Abstract

The formose reaction is often cited as a prebiotic source of sugars and remains one of the most plausible forms of autocatalysis on the early Earth. Herein, we investigated how cyanamide and 2-aminooxazole, molecules proposed to be present on early Earth and precursors for nonenzymatic ribonucleotide synthesis, mediate the formose reaction using HPLC, LC-MS and ^1^H NMR spectroscopy. Cyanamide was shown to delay the exponential phase of the formose reaction by reacting with formose sugars to form 2-aminooxazole and 2-aminooxazolines thereby diverting some of these sugars from the autocatalytic cycle, which nonetheless remains intact. Masses for tetrose and pentose aminooxazolines, precursors for nucleotide synthesis including TNA and RNA, were also observed. The results of this work in the context of the chemoton model are further discussed. Additionally, we highlight other prebiotically plausible molecules that could have mediated the formose reaction and alternative prebiotic autocatalytic systems.

## Abstract 2

The synthesis of nucleotide precursors fuelled by the formose reaction, one of the most plausible forms of autocatalysis on the early Earth, is demonstrated, and the relevance to the chemoton model is discussed.†

## Graphical abstract

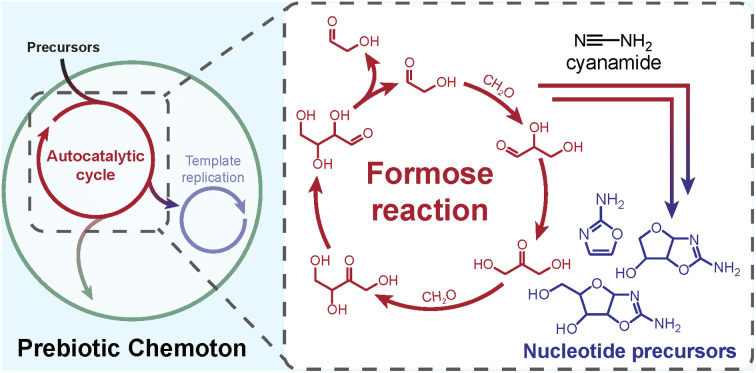

## Introduction

Tibor Gánti's theoretical model of the chemoton^1^ describes a minimal compartmentalized system capable of growth, division, and evolution that serves as a heuristic model for the generation of a minimal cell. At the heart of the chemoton is a self-sustaining autocatalytic system capable of providing the substrates for the other two required autocatalytic cycles, which afford the template-directed synthesis of a genetic polymer and the production of components for the compartment, *e.g.*, lipids. While these autocatalytic systems have been investigated independently^2,3^ in the context of prebiotic chemistry, for example, nonenzymatic template-directed nucleotide replication from activated mononucleotides,^4–9^ studies for how these autocatalytic systems could be integrated together^10^ remain few and far between. The present work herein demonstrates an example of how an autocatalytic reaction network, *i.e.*, the formose reaction, can fuel the synthesis of nucleotide precursors, and that the autoamplification kinetics of the former are intertwined with the kinetics of the latter.

The formose reaction, discovered by Butlerow,^11^ remains one of the most plausible autocatalytic reactions on early Earth as a result of the proposed abundance of the necessary feedstocks and reaction conditions.^12–15^ The formose reaction is often cited as a source of ribose for the prebiotic synthesis of ribonucleotides, a key ingredient for the RNA world,^16–20^ although not without criticism.^21,22^ As “messy chemistry” and “metabolism-first” approaches^23–27^ receive increasingly more attention within the prebiotic chemistry community, the diversity of formose products and the robustness of the reaction under different conditions have become renewed topics of interest. For example, Trapp and coworkers^28,29^ demonstrated a nonaqueous (solid state) formose reaction accelerated by mechanochemical processes. The authors also showed that the minerals incorporated as catalysts during the milling process lead to changes in product distributions. Recent report from the Huck group^30,31^ used a continuously stirred-tank reactor to elucidate how different combinations of potentially relevant environmental factors (*e.g.*, formaldehyde concentrations, initiator sugar identity, and NaOH : CaCl_2_ ratios) activate different reaction mechanisms and pathways.

**Scheme 1:**
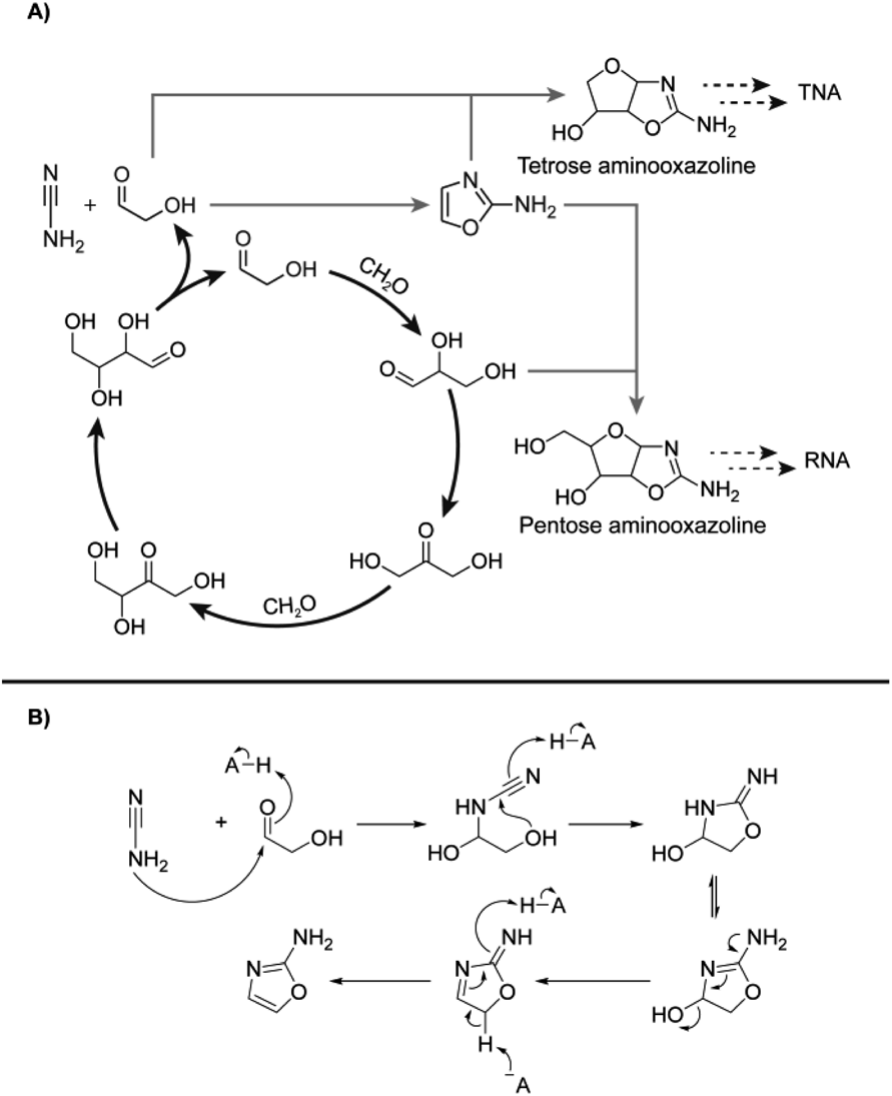
(A) Initially proposed reaction scheme involving Breslow's autocatalytic cycle^32^ (black) coupling with RNA and TNA nucleotide synthesis proceeding through 2-aminooxazole (2-NH_2_Ox) previously demonstrated by Sutherland and coworkers^51^ (grey dashed). (B) Mechanism of 2-NH_2_Ox synthesis from glycolaldehyde and cyanamide under general-base catalysed conditions.^51^

The autocatalytic nature of the formose reaction was described by Breslow^32^ (Scheme 1A, black arrows) and starts with the aldol addition between glycolaldehyde and formaldehyde (CH_2_O) to form glyceraldehyde. This C_3_ aldose isomerises to the ketose, dihydroxyacetone. A subsequent aldol addition with formaldehyde produces C_4_ ketotetroses. These ketotetroses isomerise into aldose stereoisomers, which undergo retroaldol fragmentation^30,33–35^ into two molecules of glycolaldehyde. While the complexity of the formose reaction pathways and product distributions have been extensively elaborated upon,^30,34,36–44^ Breslow's model^32^ serves as a simple demonstration of its autocatalytic nature, although more complex cycles containing higher-order sugars are also possible.^30,33,34,38,39^

The kinetics of the formose reaction can be divided into three phases. The lag phase,^22^ characterised by a period of little formaldehyde consumption, ends once glycolaldehyde and higher-order sugars have accumulated past a critical concentration. At the onset of the exponential phase, formaldehyde is rapidly consumed by aldol additions to produce higher-order sugars.^37^ These sugars then undergo retroaldol reaction, forming two additional aldol nucleophiles for every one fragmentation, creating a positive feedback loop. When formaldehyde is depleted, also known as the yellowing point,^22^ the reaction transitions to the degradation phase, which is characterised by the development of a yellow colour and a complex mixture of oligomeric products.

It is unlikely, however, that formaldehyde and other simple sugars existed as neat mixtures on early Earth. Such mixtures likely co-existed with other plausibly abundant prebiotic molecules. Yet, little is known how such molecules affect formose reaction kinetics and product distributions. For example, cyanamide (NH_2_CN) is one such simple molecule derivable prebiotically from hydrogen cyanide,^45,46^ and is known as a reagent for prebiotic chemical activation,^47,48^ production of 2-aminoimidazole^24,46,49^ and nucleotide precursor synthesis^50,51^ as well as the synthesis of arginine.^52^

Herein, the addition of cyanamide was shown to commandeer the formose reaction for nucleotide precursor synthesis, observable through changes in kinetics and product distributions. Cyanamide was investigated herein due to its established relevance to threo- and ribonucleotide synthesis^51,53–57^ and prebiotic plausibility.^24,45,46^ The effects of cyanamide and 2-aminooxazole (2-NH_2_Ox), its cyclisation product with glycolaldehyde, on formose reaction kinetics were determined *via* time course experiments monitored by high-performance liquid chromatography (HPLC), 1D proton nuclear magnetic resonance (^1^H NMR) spectroscopy and liquid chromatography-high resolution (QToF) mass spectrometry (LC-MS). It was observed that cyanamide has an inhibitory effect on formose reaction kinetics while 2-NH_2_Ox does not, and that autocatalytic sugar synthesis can be effectively commandeered for nucleotide precursor production.

## Results

The reaction of cyanamide with glycolaldehyde to form 2-NH_2_Ox, an intermediate in the Powner-Sutherland pathway for nucleotide synthesis^51^ has been well-characterised^51,53^ (see Scheme 1B). We initially hypothesised that cyanamide would react with glycolaldehyde, as well as other Breslow cycle intermediates, thereby disrupting the autocatalytic cycle, inhibiting the onset of the exponential phase, while synthesising 2-NH_2_Ox and perhaps other higher-order derivatives (Scheme 1A, grey arrows), *e.g.*, tetrose and pentose aminooxazolines.

**Fig. 1:**
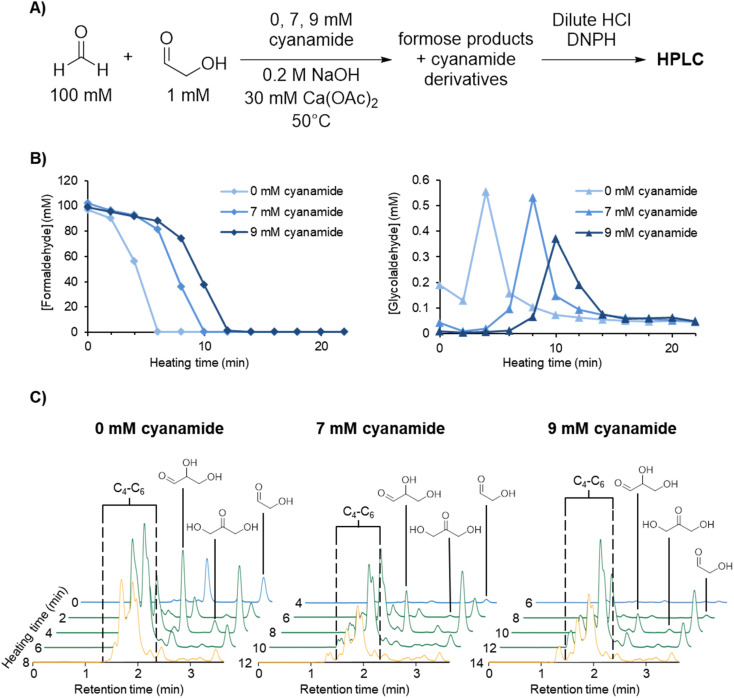
(A) Experimental scheme for formose reaction experiments containing 0, 7, and 9 mM initial cyanamide analysed by HPLC (see Methods for more details). (B) [CH_2_O] and [glycolaldehyde] over time in the formose reaction experiments outlined above based on the average of triplicates (see ESI Fig. S9–S11†). (C) Extracted HPLC chromatograms at 360 nm absorbance at selected timepoints for formose reaction experiments containing 0, 7, and 9 mM initial cyanamide (see ESI Fig. S12–S14† for all timepoints). Hemiaminal formation may lower the aldehyde concentrations during the reaction (see ESI Fig. S36 and S37† and Discussion). Chromatograms in the lag phase are coloured blue, exponential phase green, and degradation phase yellow. The intensity of the sugar peaks have a general tendency to decrease with increasing cyanamide concentrations included initially, suggesting that cyanamide is serving as a sink for formose sugars, hence inhibiting the positive feedback.

Formose reaction mixtures containing 100 mM CH_2_O, 1 mM glycolaldehyde, with various concentrations of cyanamide (0, 7, and 9 mM) were heated at 50 °C and monitored for 22 minutes by HPLC (Fig. 1A). Samples were collected every two minutes and placed on ice to stop the reaction. The concentrations of formaldehyde and glycolaldehyde were determined by 2,4-dinitrophenylhydrazine (DNPH) derivatisation followed by HPLC analysis monitored by UV-absorbance (190–450 nm). Although initial cyanamide concentrations >9 mM were also tested, it was difficult to achieve a consistent yellowing point between repeats, which varied from ∼1 hour to no yellowing point at all. It is hypothesised that the formose reaction, being autocatalytic, becomes highly sensitive to the initial conditions at these higher cyanamide concentrations, and so the variation in the yellowing point becomes large. One possible contributing factor lies in the competing Cannizarro reaction,^33,34,37,44^ which gives rise to a constant decrease in pH, further inhibiting the formose reaction, especially at these longer time scales.

Fig. 1B depicts the CH_2_O (left graph) and glycolaldehyde (right graph) concentration changes over time in formose reaction mixtures containing initial concentrations of 0, 7, and 9 mM cyanamide. The point at which CH_2_O consumption deviates from a linear decrease was used to determine the onset of the exponential phase. During this phase, glycolaldehyde production as measured by concentration reached its highest point, while CH_2_O concentration plummeted. This depletion of CH_2_O transitions the reaction from the exponential phase to the degradation phase.

The simultaneous consumption of formaldehyde and glycolaldehyde production followed by glycolaldehyde degradation after the yellowing point is consistent with trends observed in literature.^37,58^ The discrepancy between the theoretical glycolaldehyde concentration before heating (1 mM) and the observed values (0.01–0.2 mM) can be explained by glycolaldehyde participating in aldol additions, *e.g.*, with CH_2_O, as well as reaction with cyanamide immediately after initial preparation of the solution prior to heating.

**Fig. 2:**
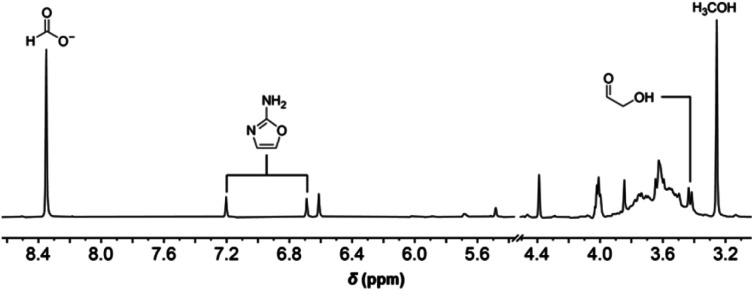
Partial ^1^H NMR spectrum of the formose reaction containing 7 mM initial cyanamide after 22 minutes of heating recorded in 10% D_2_O at pH 6.7 with phosphate buffer (see ESI Fig. S17–S19† for comparison with standards).

It was observed that the onset of the exponential phase is delayed with increasing cyanamide concentrations. At an initial concentration of 9 mM, the maximum concentration of glycolaldehyde detected during the exponential phase was also noticeably lower than reactions initially containing 7 mM or no cyanamide. This extension of the lag phase is attributed to cyanamide serving as a sink for glycolaldehyde and other sugars in the formose reaction (see Fig. 1C and ESI Fig. S15 and S16†). By preventing glycolaldehyde and larger carbohydrates from participating in autocatalytic cycles, cyanamide provides an inhibitory mechanism. The products of this inhibition are 2-NH_2_Ox alongside other oxazole and oxazoline derivatives yielded by reaction of cyanamide with other formose intermediates (*e.g.*, glyceraldehyde, tetroses, and pentoses), which were observed by LC-MS (see ESI Fig. S22–S29†). The formation of 2-NH_2_Ox at the end of the experiment (22 minutes) was also confirmed with ^1^H NMR spectroscopy (see Fig. 2 and ESI Fig. S17–S19†).

To understand whether 2-NH_2_Ox was synthesised from the initial glycolaldehyde or that produced by the formose reaction, the experiment with 7 mM cyanamide was repeated with ^13^C-labelled CH_2_O and analysed using LC-MS (ESI Fig. S20 and S21†). Before heating, only unlabelled 2-NH_2_Ox is observed. After heating started, the concentration of doubly-^13^C-labelled 2-NH_2_Ox quickly increased. The rate of production is highest during the exponential phase (6–10 minutes) and slowed down as formaldehyde is depleted (10 minutes). By the end of the observation window (22 minutes), the concentration of the doubly-^13^C-labelled species (*m*/*z*_obs_ 87.0456) was ∼1 mM (∼14% yield with respect to cyanamide), approximately four times that of the unlabelled species (*m*/*z*_obs_ 85.0396, ∼3.4% yield with respect to cyanamide). This observation indicates that the majority of 2-NH_2_Ox was made from formose-produced glycolaldehyde, which became the dominant product after the exponential phase started. Singly-^13^C-labelled 2-NH_2_Ox was also observed but only in trace amounts.

**Fig. 3:**
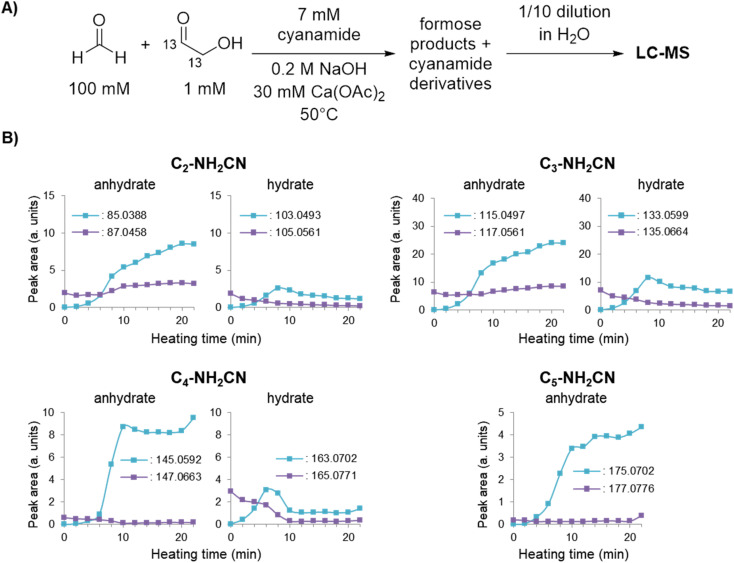
(A) Experimental scheme for formose reaction experiments containing 7 mM cyanamide analysed by LC-MS (see Methods for more details). (B) Peak areas of cyanamide adducts with C_2–5_ sugars containing 0 or 2 ^13^C isotopes based on the average of extracted ion chromatograms (EICs) taken in triplicates over time (see ESI Fig. S22–S28† for EICs).

To provide secondary confirmation that the majority of 2-NH_2_Ox is produced from formose-derived glycolaldehyde, the formose reaction with 7 mM cyanamide was repeated but with 1,2-^13^C_2_-glycolaldehyde and unlabelled CH_2_O (Fig. 3A); LC-MS was used to monitor the carbohydrate-cyanamide adducts (Fig. 3B and ESI Fig. S22†). Here, the trend previously observed for 2-NH_2_Ox was reversed, *i.e.*, the concentration of unlabelled 2-NH_2_Ox (*m*/*z*_obs_ 85.0388) overtook the doubly-^13^C-labelled species (*m*/*z*_obs_ 87.0458) after the onset of the exponential phase, the concentration of which did not increase considerably throughout the experiment. The LC-MS data was also monitored for the 2-NH_2_Ox hydrate (Fig. 3B and ESI Fig. S23†), the intermediate in 2-NH_2_Ox synthesis (Scheme 1B), the growth and decrease of which is consistent with the synthetic mechanism. The concentration of the doubly-^13^C-labelled (*m*/*z*_obs_ 105.0561) 2-NH_2_Ox hydrate was highest at the start of the reaction and decreased significantly as the reaction progressed. Meanwhile, the concentration of the unlabelled 2-NH_2_Ox hydrate (*m*/*z*_obs_ 103.0493), which was not detected before heating, began to rise at the same time as the increased glycolaldehyde production during the exponential phase and subsequently dropped at the end of it. The reaction mechanism between cyanamide and glycolaldehyde, which first yields the 2-NH_2_Ox hydrate that then undergoes dehydration to yield 2-NH_2_Ox, is consistent with these LC-MS results.

**Scheme 2:**
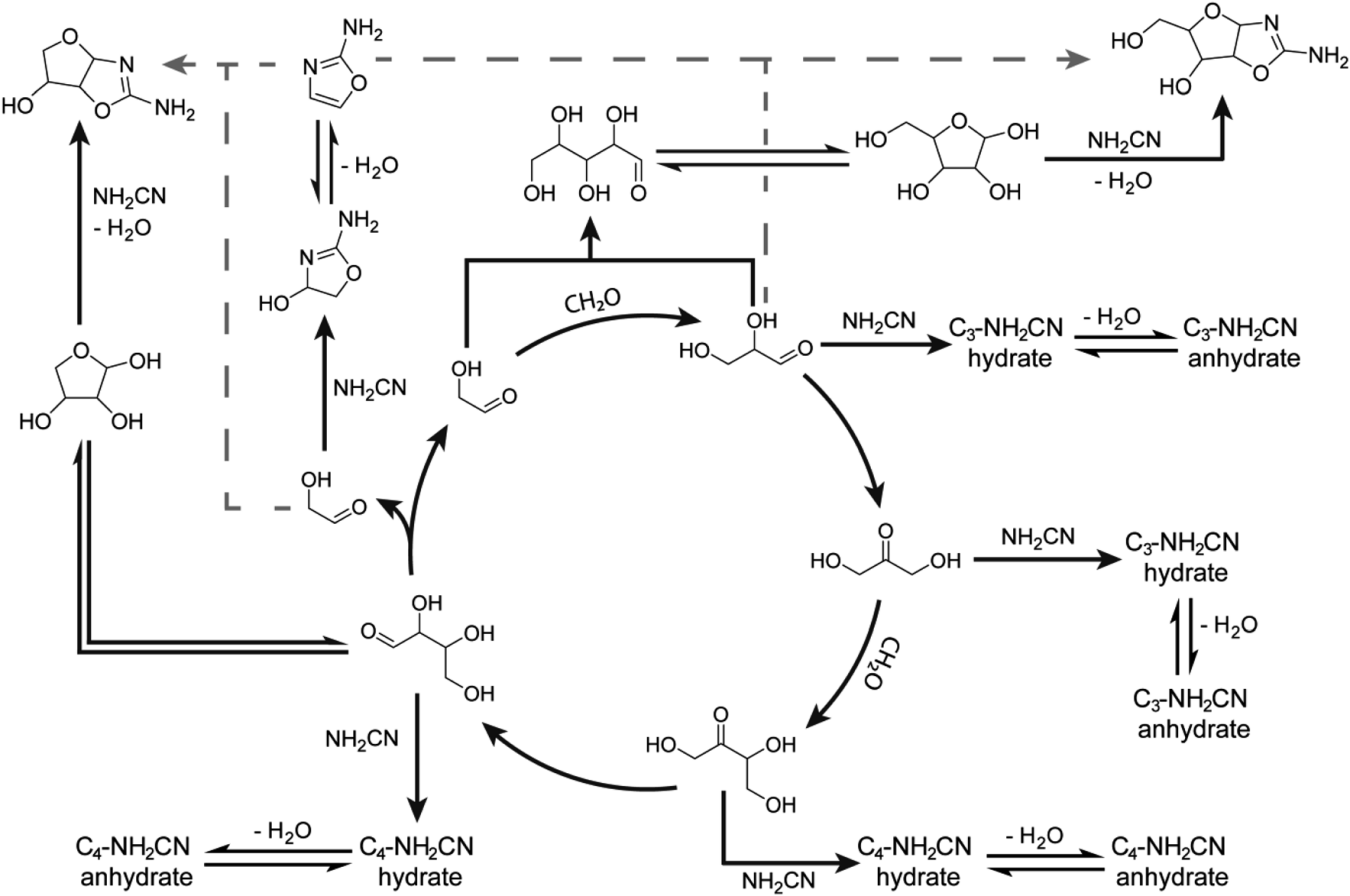
Some pathways for the proposed reaction network of formose intermediates and cyanamide based on the autocatalytic cycle proposed by Breslow^32^ and experiments herein. It is hypothesised that the formation of tetrose and pentose aminooxazolines *via* the addition of 2-NH_2_Ox with glycolaldehyde and glyceraldehyde (gray dashed arrows), respectively, is likely not the dominant pathway as originally hypothesised in Scheme 1. See ESI Table S3† for more proposed structures. Note, that although the Breslow autocatalytic cycle is shown for simplicity, the full reaction network is much more complex; see ref. 30, 41 and 42.

To demonstrate that the mechanism by which cyanamide delays the exponential phase is not limited to the removal of glycolaldehyde but also other intermediates in the Breslow cycle, we also looked for the products of cyanamide reacting with C_3–5_ sugars. As shown in Fig. 3B (see also ESI Fig. S24–S28†), the changes in concentration of cyanamide adducts with C_3_ and C_4_ sugars resembled that of the 2-NH_2_Ox hydrate, *i.e.*, the concentrations of the doubly-^13^C-labelled species (*m*/*z*_obs_ 135.0664 for C_3_, and 165.0771 for C_4_) exhibited gradual decreases while the unlabelled species (*m*/*z*_obs_ 133.0599 for C_3_ and 163.0702 for C_4_) started undetectable, reached their maximum during the exponential phase, and decreased afterwards. The concentrations of dehydrated cyanamide adducts with C_3–5_ sugars also followed similar patterns to that of 2-NH_2_Ox, *i.e.*, the doubly-^13^C-labelled species (*m*/*z*_obs_ 117.0561, 147.0663, 177.0776) did not show appreciable change, while the unlabelled species (*m*/*z*_obs_ 115.0497, 145.0592, and 175.0702) displayed significant growth, especially at the onset of the exponential phase. The proposed pathways for the production of these adducts are summarised in Scheme 2. It is important to note, the *m*/*z*_obs_ signals of 145.0592 and 175.0702 are consistent with those of tetrose and pentose aminooxazolines, threo-, ribo- and arabino-isomers of which serve as precursors for prebiotic TNA and RNA nucleotides.^51,53–57^ However, the extracted ion chromatograms for these *m*/*z* signals showed multiple peaks, suggesting a complex mixture of isomers. Collision-induced fragmentation (MS/MS) was carried out to test whether the *m*/*z*_obs_ = 175.0702 signals arise from pentose aminooxazolines. The tandem mass spectrum recorded on this ion at the end of the observation window (22 minutes) showed the same fragments in comparison to the MS/MS recorded for synthesised arabino-, ribo- and xylo-aminooxazoline standards (ESI Fig. S6–S8†), suggesting the formation of one or more of these pentose aminooxazoline stereoisomers in the formose reaction (ESI Fig. S29†).

**Fig. 4:**
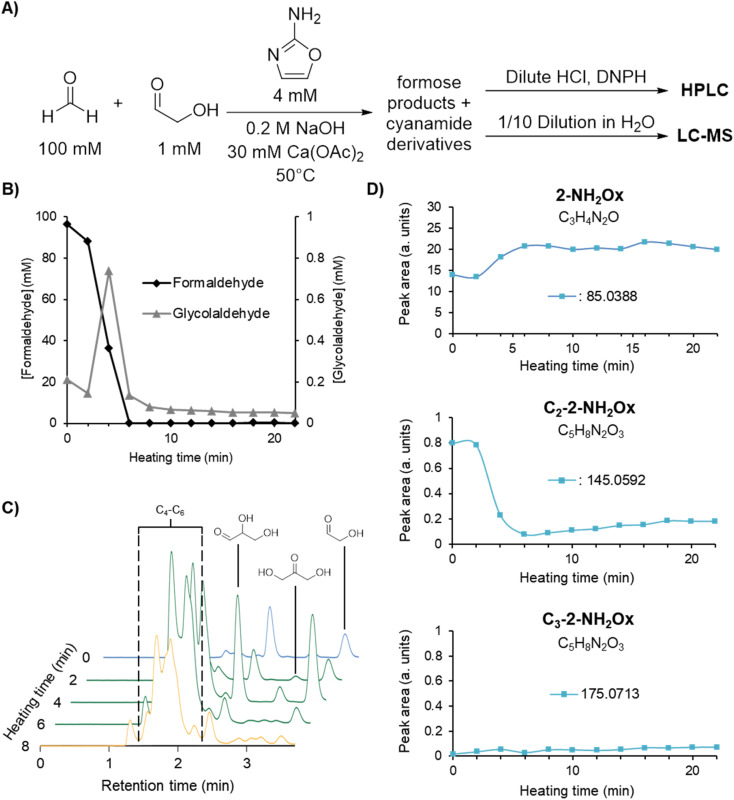
(A) Experimental procedure for formose reaction experiments containing 4 mM initial 2-NH_2_Ox analysed by HPLC and LC-MS (see Methods for more details). (B) [CH_2_O] and [glycolaldehyde] over time in formose reaction experiments outlined above based on the average of triplicates of the HPLC data (see ESI Fig. S30†). (C) Extracted HPLC chromatograms at 360 nm absorbance at selected timepoints (see ESI Fig. S31† for all timepoints). Hemiaminal formation may lower the aldehyde concentrations during the reaction (see ESI Fig. S36 and S37† and Discussion). Chromatograms in the lag phase are coloured blue, exponential phase yellow, and degradation phase green. (D) Peak areas of 2-NH_2_Ox and 2-NH_2_Ox adducts with CH_2_O and glycolaldehyde are based on the average of LC-MS EICs taken in triplicate over time (see ESI Fig. S32–S34† for EICs).

To test the impact of 2-NH_2_Ox as a potential inhibitor, the formose reaction was repeated, replacing cyanamide with 4 mM 2-NH_2_Ox (Fig. 4A), which is ∼4 times the 2-NH_2_Ox concentration yielded at the end of the formose reaction with 7 mM cyanamide (ESI Fig. S20†). 2-NH_2_Ox was hypothesised to also be capable of extending the lag phase due to its reaction as a carbon-centered nucleophile with sugars to form aminooxazoline derivatives among other products.^51,53^ Similar to previous experiments, the timepoints were analysed using HPLC and LC-MS (Fig. 4B–D). The changes in CH_2_O and glycolaldehyde concentrations displayed in Fig. 4B resemble that of the formose reaction without cyanamide (Fig. 1B). This observation suggests that 2-NH_2_Ox does not have a significant effect on the kinetics of the reaction, while the HPLC chromatograms displayed in Fig. 4C also reveal that the product distributions with and without 2-NH_2_Ox (Fig. 1C, the 0 mM cyanamide plot) are comparable.

To gain insight about the mechanism of aminooxazoline production, we also monitored *m*/*z* values consistent with tetrose and pentose aminooxazolines in the LC-MS data. It was initially expected for these signals to increase as a result of the excess 2-NH_2_Ox included initially, which ought to react with glycolaldehyde and glyceraldehyde to produce their respective aminooxazoline derivatives. Contrary to these expectations, the signal for *m*/*z*_obs_ 145.0592 was very weak (Fig. 4D and ESI Fig. S33†) while *m*/*z*_obs_ 175.0702 was negligible (Fig. 4D and ESI Fig. S34†). It was previously observed by Sutherland and coworkers that glyceraldehyde is unstable in basic pH, which prevents the assembly of pentose aminooxazolines.^53^ This finding suggests that the signals at *m*/*z*_obs_ 145.0592 and 175.0702 previously detected in the 7 mM initial cyanamide experiment arise from tetrose and pentose aminooxazolines albeit an alternative pathway, *i.e.*, the one demonstrated by Orgel and coworkers^50^ where the tetro- and pentofuranose sugars react with cyanamide to form their respective aminooxazoline products (Scheme 2).

## Discussion

The results herein demonstrate that the addition of cyanamide integrates nucleotide precursor synthesis with the autocatalytic formose reaction. Furthermore, the two processes do not simply co-exist, but rather, HPLC and LC-MS data (Fig. 1 and 2) revealed them to be kinetically coupled, a characteristic shared by many of life's chemical processes.^59^ As shown in Scheme 2, the onset of the formose exponential phase increases the production of cyanamide adducts with formose intermediates, including 2-NH_2_Ox, tetrose- and pentose-aminooxazolines. At the end of the exponential phase, production starts to plateau and the concentrations of transient hydrated species subsequently drop, likely as they convert to their corresponding anhydrates. In essence, cyanamide is capable of “peeling off” excess sugars produced by the formose reaction without completely shutting down autocatalysis. In light of these results, this work serves as a useful example of a prebiotically plausible autocatalytic cycle that fuels the synthesis of intermediates required for the production of genetic material. While the highly alkaline conditions employed herein are incompatible with nucleic acid base-pairing and hydrolytic stability, it has been shown that the formose reaction can occur under milder pH conditions,^58^ although at higher temperatures (200 °C) and pressures (100 bar).

While providing useful insights, several challenges still need to be addressed in order to fully realize the chemoton model in this context. First is the selectivity, or lack thereof, of cyanamide reactivity with other formose intermediates. In the context of prebiotic nucleotide synthesis, the cyanamide-formose products observed, excluding 2-NH_2_Ox, threo-, ribo- and arabino-aminooxazolines, might be considered as waste products which may interfere with further downstream reactions. However, it has also been proposed that the waste of one autocatalytic system can activate and/or serve as “food” for another, giving rise to networks of sequentially triggered autocatalytic subsystems.^60^ The activation or deactivation of autocatalytic motifs in such a network could give rise to different heritable states and provide the basis for chemical evolution, however, the prediction of (coupled) autocatalytic networks is a notoriously difficult problem, and their emergence in a prebiotic context has been criticised as improbable.^61^ Further research is required.

Alternatively, encapsulation of the reaction network within a selectively permeable membrane that could allow for the accumulation of useful intermediates while removing unwanted waste is also a potential solution. For example, Sacerdote and Szostak^62^ showed that a range of model fatty acid or phospholipid membranes exhibit enhanced permeability for ribose over other sugars of similar size. The authors noted that the preference for ribose assimilation may have potentially contributed to the emergence of the RNA world.^62^

Another method to accumulate useful products is to phosphorylate them to prevent their diffusion through the membrane. The addition of a negatively charged phosphate group is how modern cells trap glucose in the first step of glycolysis. A potential mechanism for aqueous prebiotic phosphorylation involves the use of diamidophosphate (DAP), a di-nitrogenous analogue of orthophosphate which could have formed on the early Earth, *e.g.*, by reacting aqueous ammonia with iron phosphide or phosphorus(v) oxide.^63^ DAP has been shown to phosphorylate a wide variety of biological building blocks including nucleo(s/t)ides, amino acids, and lipid precursors under mild conditions.^64–67^

Another issue is the fact that that under the experimental conditions tested herein, 2-NH_2_Ox does not contribute significantly to tetrose and pentose aminooxazoline synthesis. This observation was attributed mechanistically to the relative instability of glycolaldehyde and glyceraldehyde, which more rapidly undergo aldol additions instead of cycloaddition with 2-NH_2_Ox under alkaline pH. We propose that regular fluctuations in pH between alkaline and neutral conditions may afford periods of autocatalytic sugar and 2-NH_2_Ox production followed by 2-NH_2_Ox-dependent synthesis of tetrose and pentose aminooxazolines, respectively. Such fluctuating conditions of pH could have been realized in a terrestrial hydrothermal field where hot springs of highly varied conditions of pH exist in close proximity and frequently mix with one another.^68,69^

A crucial feature of the chemoton model implied in this discussion is the complete synthesis of (activated) nucleotides that can be eventually exploited in autocatalytic (template-directed) genetic polymer replication. Following the production of aminooxazolines, the next step in the Powner-Sutherland nucleoside synthesis involves the cycloaddition of cyanoacetylene with tetrose or pentose aminooxazolines to form anhydronucleosides, which serve as common intermediates for both purine and pyrimidine nucleo(s/t)ides.^51,54–57^ Arabino- and threo-anhydronucleosides can be converted to pyrimidine nucleotides *via* urea-mediated phosphorylation.^51,57^ Ribo-anhydronucleosides can undergo addition with 8-mercaptoadenine followed by photoreduction in the presence of sulfite/bisulfite to form purine ribo- and deoxyribonucleosides.^55,56^ Given sufficient activation chemistry, the resulting racemic mixture of DNA, RNA, and TNA nucleotides may give rise to genetic polymers with heterogenous sugar backbones^70^ that could potentially serve as the basis for nonenzymatic template-directed replication in the chemoton. While all these prebiotic nucleotide syntheses have been reported, whether or not such chemistry can be effectively coupled to the autocatalytic formose reaction and encapsulated in the context of a chemoton model is yet to be determined.

In this work, we have demonstrated the effect of cyanamide on the formose reaction, however, other prebiotically plausible molecules that could mediate the formose reaction in interesting ways deserve highlight. Racemic alanine was previously demonstrated to catalyse aldol additions in slightly acidic conditions, forming not only higher order aldoses and ketoses but also pyruvate, pyruvaldehyde, and glyoxal.^71^ The reaction between cyanide with aldehydes and ketones form cyanohydrins, which can be highly stable (*e.g.*, for glycolonitrile, *K* = 10^6^ M^−1^).^72,73^ It has been observed that cyanohydrins such as glycolonitrile can accelerate HCN oligomerisation, potentially contributing to prebiotic nucleobase synthesis.^74,75^ Given that CH_2_O is in large excess, we supposed that formaldehyde would quickly sequester any cyanide, leaving the autocatalytic cycle of the formose reaction and its kinetics relatively unaffected. Fig. S35† shows 0.1 eq of sodium cyanide has little to no effect on the timing of the formose reaction yellowing point. The produced cyanohydrins, primarily glycolonitrile, may accelerate HCN oligomerisation in a neighbouring environment, leading to nucleobase formation.

However, free HCN and/or its derivatives are necessary for cyanamide synthesis.^46,52,76^ There may be potential prebiotic scenarios that can afford cyanamide synthesis yet prevent HCN from hindering the formose reaction. For example, in one scenario, a transiently reduced atmosphere caused by exogenous delivery and impact of reduced materials leads to the atmospheric synthesis of cyanamide.^46,76^ Precipitation onto the surface introduces cyanamide to bodies of water which previously accumulated formaldehyde and other formose intermediates while the atmosphere was neutral.^77^ Another scenario involves gamma irradiation which has been previously demonstrated by Yi *et al.*^24^ Here, hydrogen cyanide serves as the feedstock for the continuous production of cyanamide as well as formaldehyde- and glycolaldehyde-derived cyanohydrins. Addition of excess CH_2_O produced through other geochemical processes^13,14,76^ may liberate enough glycolaldehyde to kickstart the formose reaction in the presence of cyanamide, assuming sufficiently basic pH and catalytic Ca^2+^ concentrations are present.

At basic pH, borate is known to complex with formose intermediates that possess *syn*-1,2-diols, which stabilises higher-order sugars (C_4_ and above) from undergoing retroaldol fragmentation, a feature which also essentially inhibits the positive feedback mechanism needed for autocatalysis.^40,42,43,78^ If these sugar–borate complexes were to undergo pH fluctuations to neutral and borate subsequently sequestered, the released sugars may be able to initiate the formose reaction once the pH increases. Hence, although the “messy” early Earth could have hosted an array of potential prebiotic molecules capable of interfering with the formose reaction, we have identified some cases where such interference could trigger downstream reaction cycles and give rise to further and potentially beneficial complexity.

Alternatives for prebiotic sugar synthesis can be found in Eschenmoser's glyoxylate scenario^79^ and the recently proposed glyoxylose reaction.^80^ The glyoxylate scenario^79^ relies on the glyoxylic acid dimer, dihydroxyfumarate, which reacts with its constituents and other compounds to produce a range of classes of biomolecules, including sugars, amino acids and pyrimidines. The glyoxylose reaction proposed by Krishnamurthy and Liotta^80^ relies on aldol additions involving glyoxylate to produce sugars and sugar acids. Although the potential for retroaldol fragmentation in both reactions suggests the possibility for autocatalysis, this has yet to be confirmed experimentally. Nevertheless, the ability to produce sugars, among other classes of molecules, and the potential for autocatalysis hint that these proposed reactions could serve the role of the central metabolic cycle in the chemoton model.

Outside sugar synthesis, other autocatalytic cycles capable of fuelling membrane synthesis and self-replication have also been investigated, most notably the reductive TCA (rTCA) cycle. The appeal of the rTCA cycle involves the production of five universal metabolites including the biological precursors for lipids and nucleotides, molecules that make up the membrane and self-replication mechanism.^59^ While the nonenzymatic rTCA cycle is autocatalytic in theory, its unfavourable kinetics prevents the cycle from being of practical use,^61^ especially in “messy” environments often associated with early Earth. Although much progress^81–85^ has been made in elucidating the prebiotic conditions necessary for various steps in the cycle, a complete cycle of sufficient turnover has not yet been demonstrated. As such, its relevance to the chemoton model remains uncertain.

## Conclusions

Herein, we demonstrate the synthesis of nucleotide precursors fuelled by an autocatalytic cycle as proposed by Tibor Gánti's chemoton model^1^ that is consistent with prebiotic chemistry. The addition of cyanamide was observed to commandeer the autocatalytic formose reaction for the production of intermediates in the nucleotide synthesis pathways proposed by Orgel *et al.*^50^ and Sutherland *et al.*^51,53^ Reaction of cyanamide and formose-derived glycolaldehyde led to the production – primarily during the exponential phase – of 2-NH_2_Ox, an intermediate in nonenzymatic nucleotide synthesis.^51,53^ Masses consistent with tetrose and pentose aminooxazolines, certain stereoisomers of which serve as the next intermediates in TNA and RNA nucleoside synthesis,^53^ respectively, were also detected in the cyanamide-mediated formose reactions. Moreover, LC-MS data revealed that the synthesis of these precursors is kinetically coupled to the production of sugars and are not simply linear chemical reactions between cyanamide and isolated formose-derived sugars. With this work, we aim to contribute to experimental studies investigating the chemoton model in the context of prebiotic chemistry as well as integrating nonlinear dynamics and prebiotic synthesis.

## Experimental

### Materials and methods

#### Materials

All experiments were conducted in 18 MΩ water processed with a Milli-Q purification system. ^13^C-labelled formaldehyde, dinitrophenylhydrazine (DNPH), glycolaldehyde, paraformaldehyde, d-xylose, d-erythrose, cyanamide, glucose monohydrate, dansyl chloride (DsCl), sodium cyanide, and calcium acetate monohydrate (Ca(OAc)_2_·H_2_O) were purchased from Sigma-Aldrich. ^13^C-labelled glycolaldehyde was purchased from ChemCruz. 2-Aminooxazole (2-NH_2_Ox) was purchased from Combi-Blocks. HPLC-grade acetonitrile was purchased from VWR International Pty Ltd. LCMS-grade acetonitrile and water were purchased from Chem-Supply Pty Ltd. All reagents were used without further purification. 37% formaldehyde stock solutions and 1 M pH 6.7 sodium phosphate buffer were prepared according to established protocols,^86,87^ and the formose stock was subsequently diluted to 1 M with Milli-Q water. DNPH was dissolved in acetonitrile (ACN) at 3 mg mL^−1^. Stock solutions of organic compounds were stored at 5 °C and were made fresh after one week. Arabino-, ribo-, and xylo-aminooxazoline were synthesised according to literature procedures.^50^

#### Formose reaction time course experiments

In order to carry out the formose reactions, two separate mixtures containing formaldehyde (mixture A) and glycolaldehyde (mixture B) were prepared separately at room temperature and then mixed (see Tables S1 and S2†). Mixture A contains CH_2_O, cyanamide, and NaOH while B contains glycolaldehyde and calcium acetate. N.B. minimal precipitation, presumably Ca(OH)_2_, was observed to occur when mixtures A and B were combined; precipitation was observed most when either [NaOH] or [calcium acetate] is high. For each experiment performed, the mixtures A and B were combined at a 1 : 1 ratio by volume (6.8 mL each) in a 15 mL centrifuge tube and mixed immediately *via* shaking and vortexing. The combined mixture was subsequently divided into 12 × 1 mL aliquots which were placed into 1.5 mL centrifuge tubes containing magnetic stirrer bars. 11 of the 12 sample tubes were continuously stirred and incubated at 50 °C while the remaining tube was placed on ice to quench the reaction as an initial timepoint. To monitor the reaction over time, every 2 minutes one of the 1 mL aliquot samples being heated at 50 °C was removed from heat and placed on ice in order to stop the reaction.

#### HPLC analysis

80 μL of each timepoint sample was transferred to an HPLC vial containing 120 μL of 0.15 M HCl for quenching. N.B. without addition of HCl prior to derivatisation, a peak (RT = 2.43 min) with UV absorption uncharacteristic of DNPH derivatisation was observed. The following derivatisation method employed is adapted from Haas *et al.*^88^ First, 800 μL of a derivatisation mixture containing 700 μL of DNPH dissolved in ACN (3 mg mL^−1^), 95 μL of neat ACN, and 5 μL of 2 M HCl was prepared and transferred to each HPLC vial. The derivatisation reaction then was allowed to proceed at room temperature for a minimum of 30 minutes before HPLC analysis.

HPLC analysis was carried out using a Shimadzu Nexera 40 Series UPLC system with PDA detector (Kyoto, Japan). An aliquot of 1 μL of each derivatised sample was injected into the HPLC and eluted with a 1 mL min^−1^ isocratic flow of 50 : 50 aqueous 0.1% formic acid (solvent A) and ACN with 0.1% formic acid (solvent B) for 20 minutes. Following each sample run was a binary gradient wash. The gradient started at 50% solvent B and stayed at 50% for 9 minutes. Solvent B was then ramped up to 90% in 3 minutes after which the concentration remained at 90% for 4 minutes. The concentration of B then returned to 50% in 30 seconds and remained at 50% for the rest of the run. The stationary phase was a Shimadzu Shim-pak GIST C18 column (5 μm particle size, 4.6 mm I.D. and 150 mm length) with the oven temperature maintained at 25 °C.

#### LC-MS

100 μL of each timepoint sample was transferred to a 1.5 mL microcentrifuge tube containing 900 μL of Milli-Q water. The formose samples were stored at 5 °C for 2–3 days to allow the cyanamide and 2-NH_2_Ox adducts with formose intermediates to reach equilibrium in the diluted condition before LC-MS analysis (see ESI Fig. S36 and S37† and Discussion).

LC-MS analysis was carried out using a Shimadzu Nexera 40 Series UPLC system connected to a Shimadzu LC-MS-9030 quadruple-time-of-flight mass spectrometer (Kyoto, Japan). A Thermo Scientific Hypercarb UPLC column (3 μm particle size, 2.1 mm I.D., 50 mm length) was used with the oven temperature maintained at 40 °C. The diluted formose samples were injected at 1 μL aliquots and eluted at 0.2 mL min^−1^ using a binary gradient made up from two solvents: (A) water + 0.1% formic acid and (B) acetonitrile + 0.1% formic acid. Each run started with 0% acetonitrile, which was maintained for 2 minutes, then increased from 0% to 50% over 4 minutes and maintained at 50% for 1.5 minutes. Solvent B was then ramped down from 50% back to 0% in 1 minute and maintained at 0% for 2.5 minutes to complete the gradient. Between each analysis, Milli-Q water (10 μL) was injected and the column was washed using the same gradient. Analytes with retention time 0.4–5 min were analysed using positive-mode MS analysis. Ions with *m*/*z* 175.0719 were further analysed *via* collision-induced disassociation MS/MS with collision energy set to 35 V.

#### ^1^H NMR spectroscopy

To quench the formose timepoints, 90 μL of 2 M HCl was added to samples before heating (initial time point) and 80 μL for samples after 22 minutes of heating. A 700 μL aliquot of these quenched formose mixtures were then transferred to 1.5 mL Eppendorf tubes containing 100 μL D_2_O and 200 μL 1 M pH 6.7 sodium phosphate buffer, resulting in a final pH of ∼7. Standards (glycolaldehyde, 2-NH_2_Ox, glyceraldehyde, methanol, and calcium acetate) were added and mixed *via* pipetting up and down. 600 μL of the final mixtures were transferred to NMR tubes for analysis.

NMR spectroscopy was performed using a Bruker Avance III HD 600 (600.16 MHz, 1H) spectrometer. 1D ^1^H NMR spectra were collected with water suppression using a presaturation pulse program (zgcpgppr). The number of scans was set to 32.

## Data availability

All the supporting experimental data is a part of ESI.†

## Author contributions

Q. P. T, R. Y., and A. C. F. designed research; Q. P. T. performed research; Q. P. T. and A. C. F. analysed data; Q. P. T. and A. C. F. wrote the paper.

## Conflicts of interest

The authors declare no conflict of interest.

## Supplementary Material

SC-014-D3SC03185C-s001
