# Supplementary material for: Towards a prebiotic chemoton – nucleotide precursor synthesis driven by the autocatalytic formose reaction
Source: Chem Sci. 2023 Aug 29;14(35):9589–99. doi: 10.1039/d3sc03185c (PMC10498504; doi:10.1039/d3sc03185c)
Supplement: SC-014-D3SC03185C-s001 [file SC-014-D3SC03185C-s001.pdf]

Electronic Supplementary Material (ESI) for Chemical Science.
This journal is © The Royal Society of Chemistry 2023

Towards a Prebiotic Chemoton – Nucleotide Precursor Synthesis Driven by the Autocatalytic Formose Reaction

Quoc Phuong Tran[ab], Ruiqin Yi[d], Albert C. Fahrenbach*[abc]

[a]School of Chemistry and [b]Australian Centre for Astrobiology, [c]UNSW RNA Institute, University of New South Wales, Sydney, NSW 2052, Australia; [d]Earth-Life Science Institute, Tokyo Institute of Technology, Tokyo 152-8550

Supplementary Information

CONTENTS

Additional Methods

1) Formose Reaction
2) HPLC Analysis
   - 1.1. Standard Curves of DNPH-Derivatised Formaldehyde and Glycolaldehyde
   - 1.2. DsCl Derivatisation of Cyanamide in Formose Samples
   - 1.3. Standard Curve of Dansyl Chloride-Derivatised Cyanamide
   - 1.4. HPLC Analysis of DsCl-Derivatised Formose Samples
3) LC-MS Analysis
   - 2.1 Sample Preparation for LC-MS Analysis
   - 2.2 Data analysis
4) ^{1}H NMR Spectroscopy
   - 3.1 Data Analysis

Experimental data

5) Standard Curves
6) Time Course Experiments of Formose Reaction with 0, 7, or 9 mM Cyanamide
7) Time Course Experiment of Formose Reaction with 4 mM 2-Aminooxazole
8) Yellowing Point Assay of the Formose Reaction with 10 mM Sodium Cyanide
9) Control Experiments and Discussion Regarding Hemiaminal Formation in Formose Reaction Samples

References

Additional Methods

1. Formose Reaction

Table S1. Composition of formaldehyde-containing reaction mixtures A. To investigate each condition (column 1), respective components were added in order from left to right into a 15 mL centrifuge tube. Following the addition of 1 M NaOH, the mixtures were vortexed until homogenous (~ 4 seconds).

	1 M CH_2O (μL)	MQ water (μL)	1 M cyanamide (μL)	1 M 2-NH_2Ox (μL)	1 M NaOH (μL)
**Effect of [cyanamide]**					
0 mM cyanamide	1400	2800	0	0	2800
7 mM cyanamide	1400	2702	98	0	2800
9 mM cyanamide	1400	2674	126	0	2800
**Effect of [2-NH_2Ox]**					
4 mM 2-NH_2Ox	1400	2744	0	56	2800

Table S2. Composition of glycolaldehyde-containing mixtures B. The components were added in order from left to right into a 15 mL centrifuge tube. Following the addition of 1 M $Ca(OAc)_2$, the mixtures were vortexed until homogenous (~ 4 seconds).

	0.1 M glycolaldehyde (μL)	MQ water (μL)	1 M $Ca(OAc)_2$ (μL)
**Effect of [cyanamide]**			
0 mM cyanamide	140	6440	420
7 mM cyanamide	140	6440	420
9 mM cyanamide	140	6440	420
**Effect of [2-NH_2Ox]**			
4 mM 2-NH_2Ox	140	6440	420

2. HPLC Analysis

2.1. Standard Curves of DNPH-Derivatised Formaldehyde and Glycolaldehyde

The formaldehyde stock solution was diluted to 100, 50, 25, 12.5 and 6.25 mM, and glycolaldehyde to 10, 5, 2.5, 1.25 and 0.625 mM with Milli-Q water. For the construction of standard curves, an 80 μL aliquot of each standard solution was added to 120 μL of Milli-Q water followed by 800 μL of the derivatisation mixture described above. The derivatisation reaction was allowed to proceed at room temperature for a minimum of 30 minutes before HPLC analysis as described in the main text.

2.2. DsCl Derivatisation of Cyanamide in Formose Samples

110 μL of each timepoint sample was transferred to a 1.5 mL microcentrifuge tube containing 10 μL of 2 M HCl for quenching followed by the addition 80 μL of 0.4 M Na_2CO_3/$NaHCO_3$ buffer (pH 9). The mixture was mixed by pipetting up and down after which 200 μL of 10 mg mL^{-1} DsCl in HPLC-grade acetone was added using a 500 μL glass syringe (Innovative Labor Systeme GmbH). The resulting mixture was vortexed for 1 minute. The derivatisation reaction then was heated to ~45 °C for approximately 70 minutes followed by centrifugation for 1 minute. The supernatant was then transferred to HPLC vials for analysis as described in the main text.

2.3. Standard Curve of Dansyl Chloride-Derivatised Cyanamide

A stock solution of cyanamide was serially diluted to 15, 7.5, 5, 2.5, 1.25 and 0.625 mM with Milli-Q water. For the construction of standard curves, a 110 µL aliquot of each concentration was added to 10 µL of Milli-Q water followed by 80 µL of the 0.4 M $NaHCO_3/Na_2CO_3$ (pH 9) buffer. The derivatisation reaction was heated at 45 °C for 70 minutes, and the derivatised mixtures were transferred to HPLC vials for analysis. The derivatised samples were stored at 5 °C in the autosampler to inhibit decomposition.

2.4. HPLC Analysis of DsCl-Derivatised Formose Samples

HPLC analysis was carried out using a Shimadzu Nexera 40 Series UPLC system with PDA detector (Kyoto, Japan). An aliquot of 1 µL of each derivatised sample was injected into the HPLC and eluted with a 1 mL min^{-1} binary gradient consisting of 10 mM NaH_2PO_4 (solvent A) and neat ACN (solvent B) for 30 minutes. The gradient started at 45% B and ramped up to 80% in 14 minutes. From 14 minutes to 15 minutes, the concentration of B went from 80% to 90% and remained at 90% for 7 minutes. From 22 minutes to 23 minutes, the concentration returned to 45%. Solvent B stayed at 45% for the rest of the run. The same binary gradient was used as a wash after every sample run. The stationary phase was a Shimadzu Shim-pak GIST C18 column (5 µm particle size, 4.6 mm I.D., and 150 mm length) with the oven temperature maintained at 25 °C. The derivatised samples were stored at 5 °C in the autosampler to inhibit decomposition.

3. LC-MS Analysis

3.1 Standard Curve of 2-NH_2Ox

A stock solution of cyanamide was serially diluted to 1.25, 0.625, 0.5, 0.3125, 0.25, 0.0625 and 0.03125 mM with Milli-Q water. 100 µL of each sample was transferred to a 1.5 mL microcentrifuge tube containing 900 µL of Milli-Q water. The formose samples were stored at 5 °C for 2–3 days to allow the cyanamide and 2-NH_2Ox adducts with formose intermediates to reach equilibrium in the diluted condition before LC-MS analysis.

3.2 Data Analysis

Microsoft Excel was used to plot HPLC chromatograms. Origin 2022b was used to smoothen and plot LC-MS extracted ion chromatograms (EICs). The EICs were smoothened using an adjacent-averaging method with a window of 30 points and without weighted average.

4. ^{1}H NMR Spectroscopy

4.1 Data analysis

MestreNova (MestreLab Research, Santiago de Compostela, Spain) was used to analyse and compile NMR spectra.

5. Standards and Concentration Curves

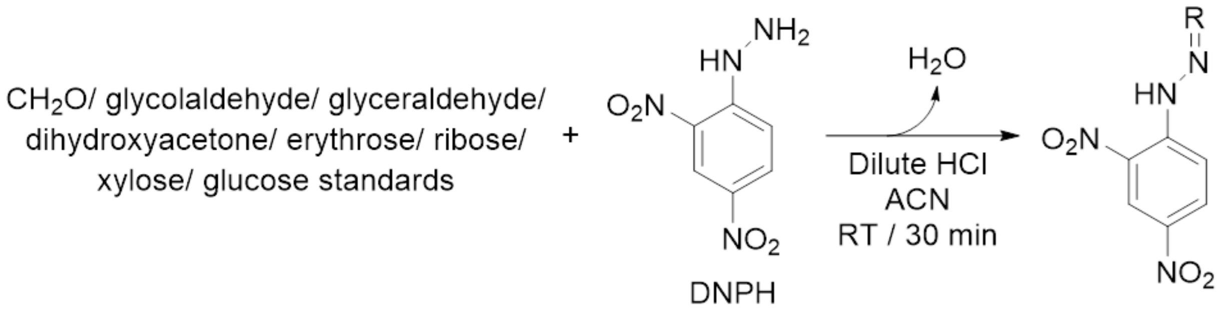

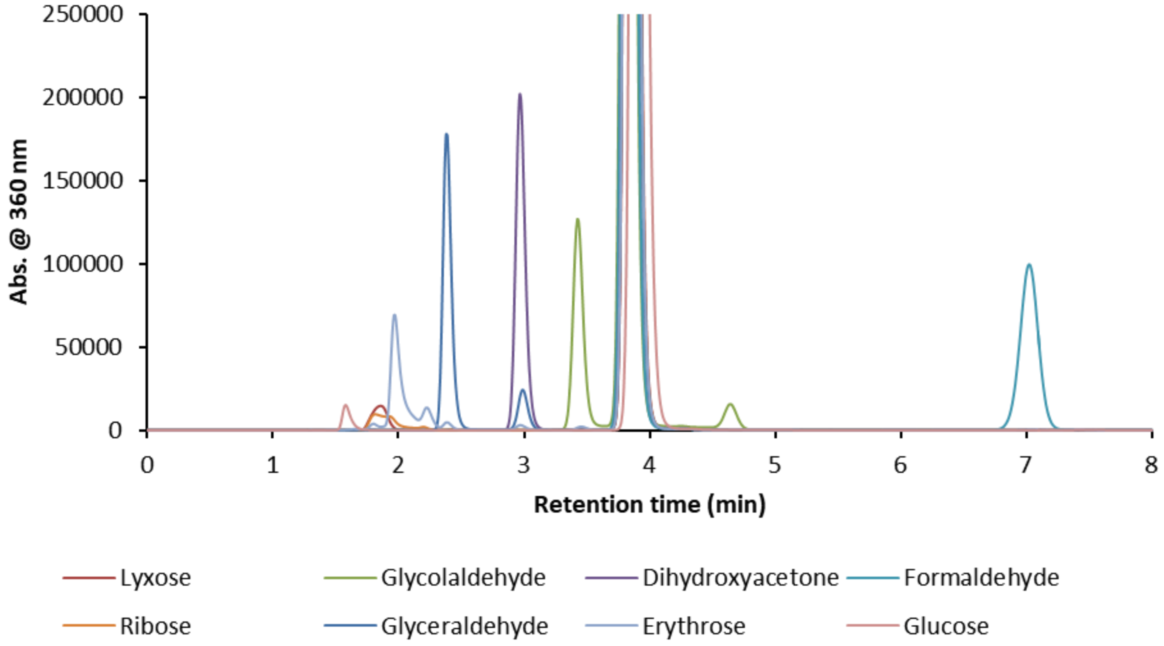

Figure S1. Overlayed HPLC chromatograms of DNPH-derivatised formose reagents and intermediates C_{1-6}.

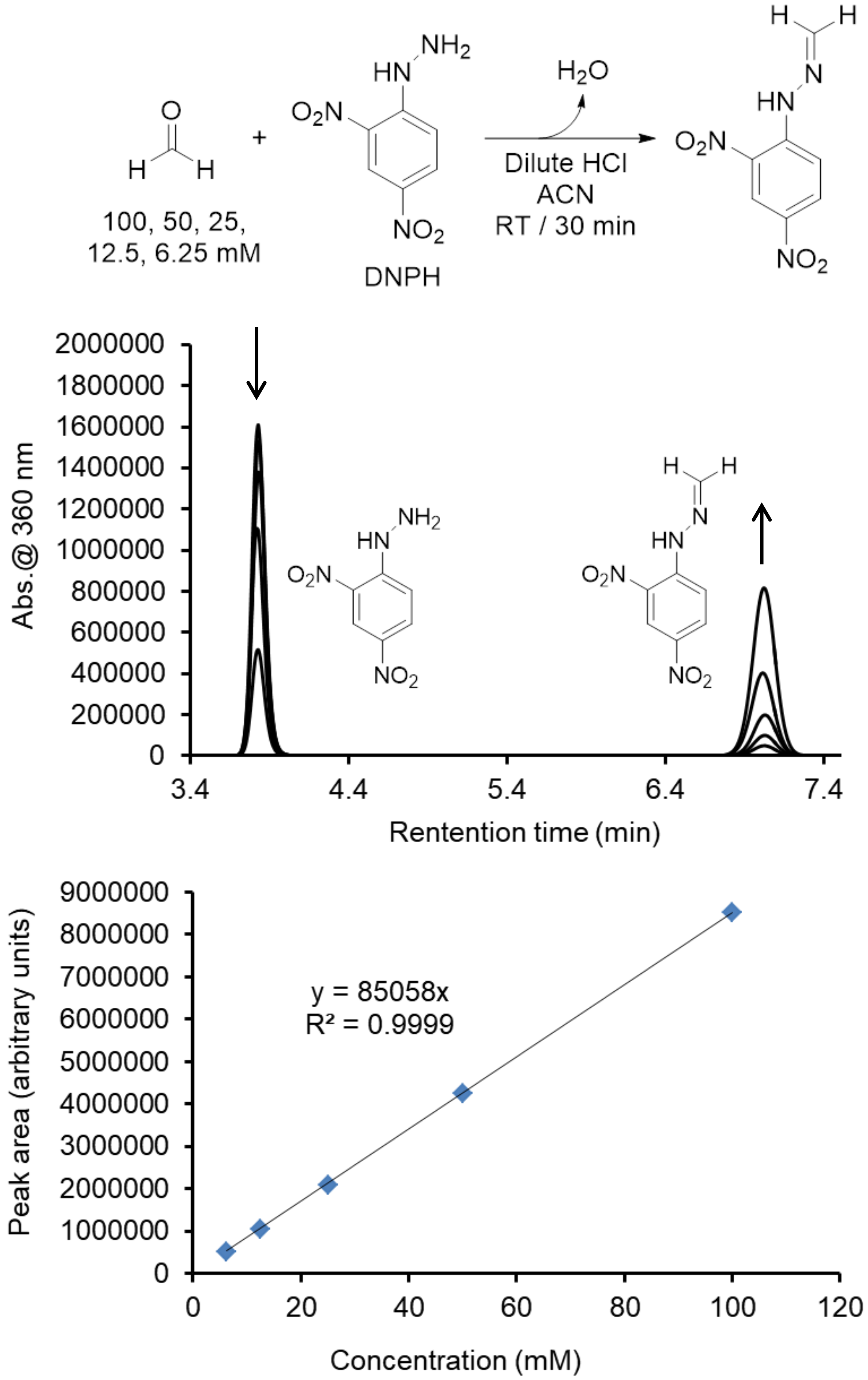

Figure S2. Standard curve of formaldehyde derivatised with DNPH determined by HPLC analysis. Top) Reaction scheme of CH_2O derivatisation. Middle) HPLC chromatograms at 360 nm showing the different concentrations of formaldehyde standards derivatised with DNPH. Bottom) Standard curve of DNPH-derivatised formaldehyde.

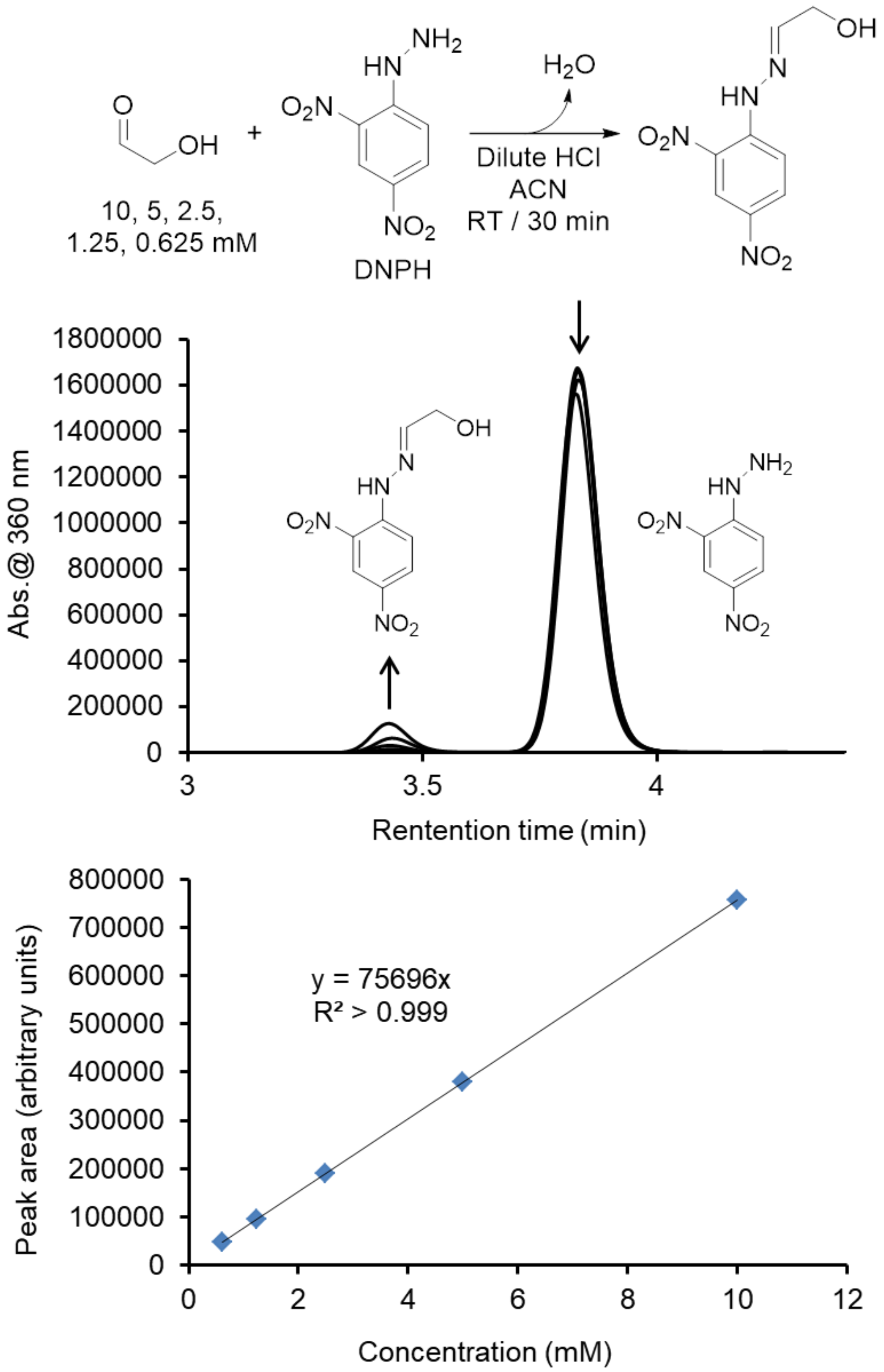

Figure S3. Standard curve of glycolaldehyde derivatised with DNPH determined by HPLC analysis. Top) Reaction scheme of glycolaldehyde derivatisation. Middle) HPLC chromatograms at 360 nm showing the different concentrations of glycolaldehyde standards derivatised with DNPH. Bottom) Standard curve of DNPH-derivatised glycolaldehyde.

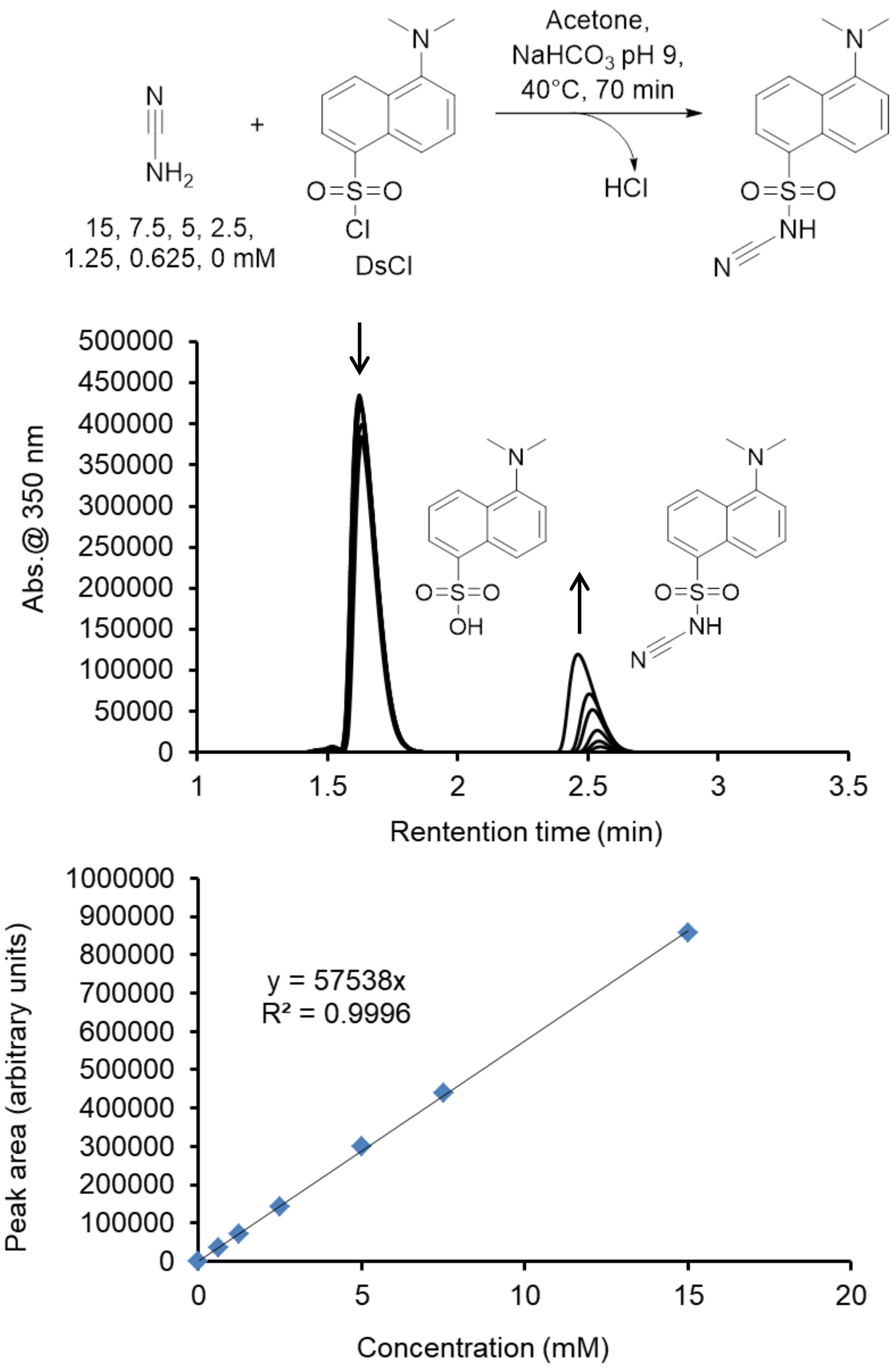

Figure S4. Standard curve of cyanamide derivatised with DsCl determined by HPLC analysis. Top) Reaction scheme of cyanamide derivatisation. Middle) HPLC chromatograms at 350 nm showing the different concentrations of cyanamide standards derivatised with DsCl. Bottom) Standard curve of DsCl-derivatised cyanamide.

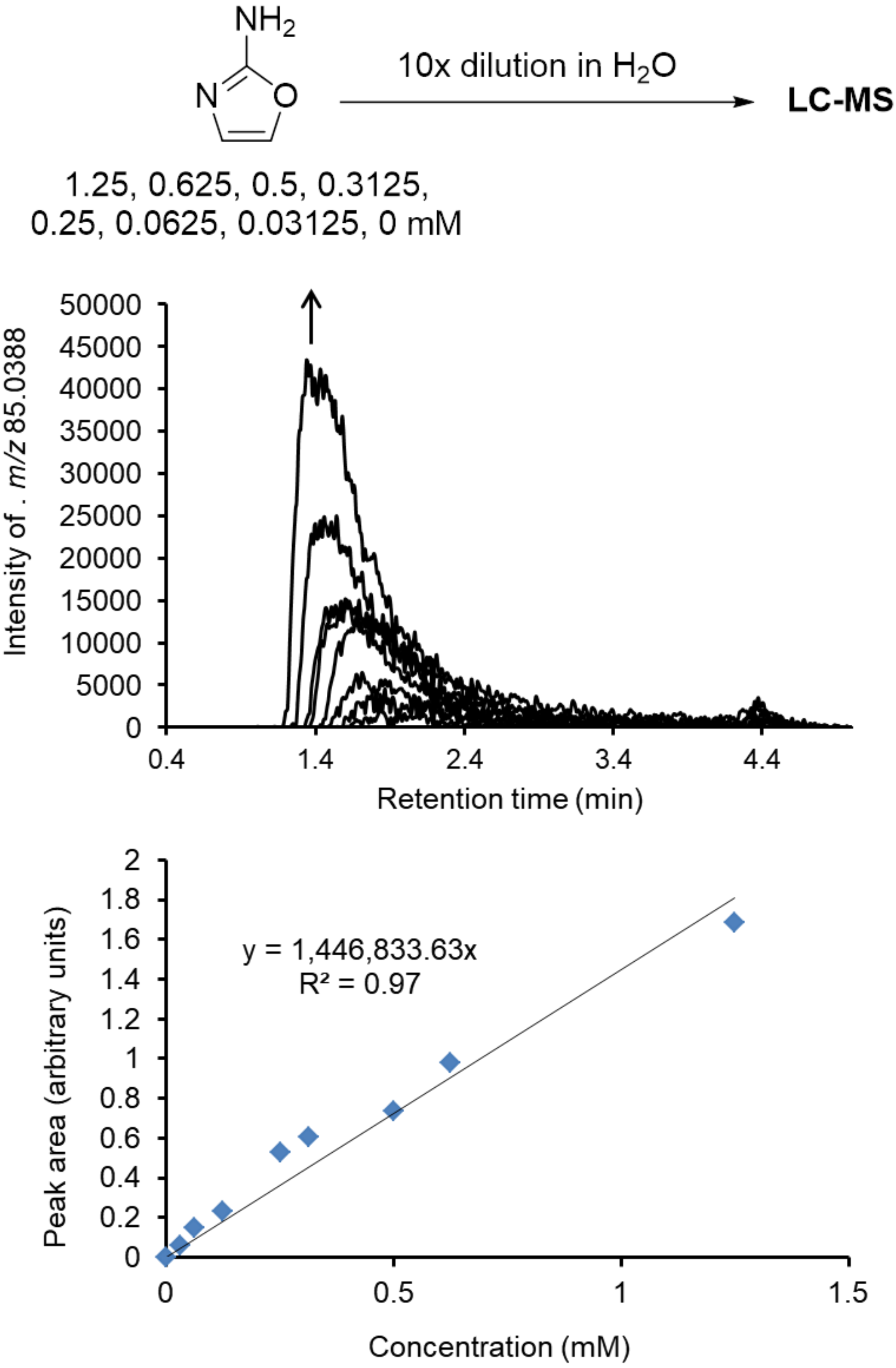

Figure S5. Standard curve of 2-NH_2Ox determined by LC-MS analysis. Top) Procedure of 2-NH_2Ox LC-MS analysis Middle) Extracted ion chromatograms of m/z_{obs} 85.0388 +/– 10 ppm showing the different concentrations of 2-NH_2Ox. Bottom) Standard curve 2-NH_2Ox.

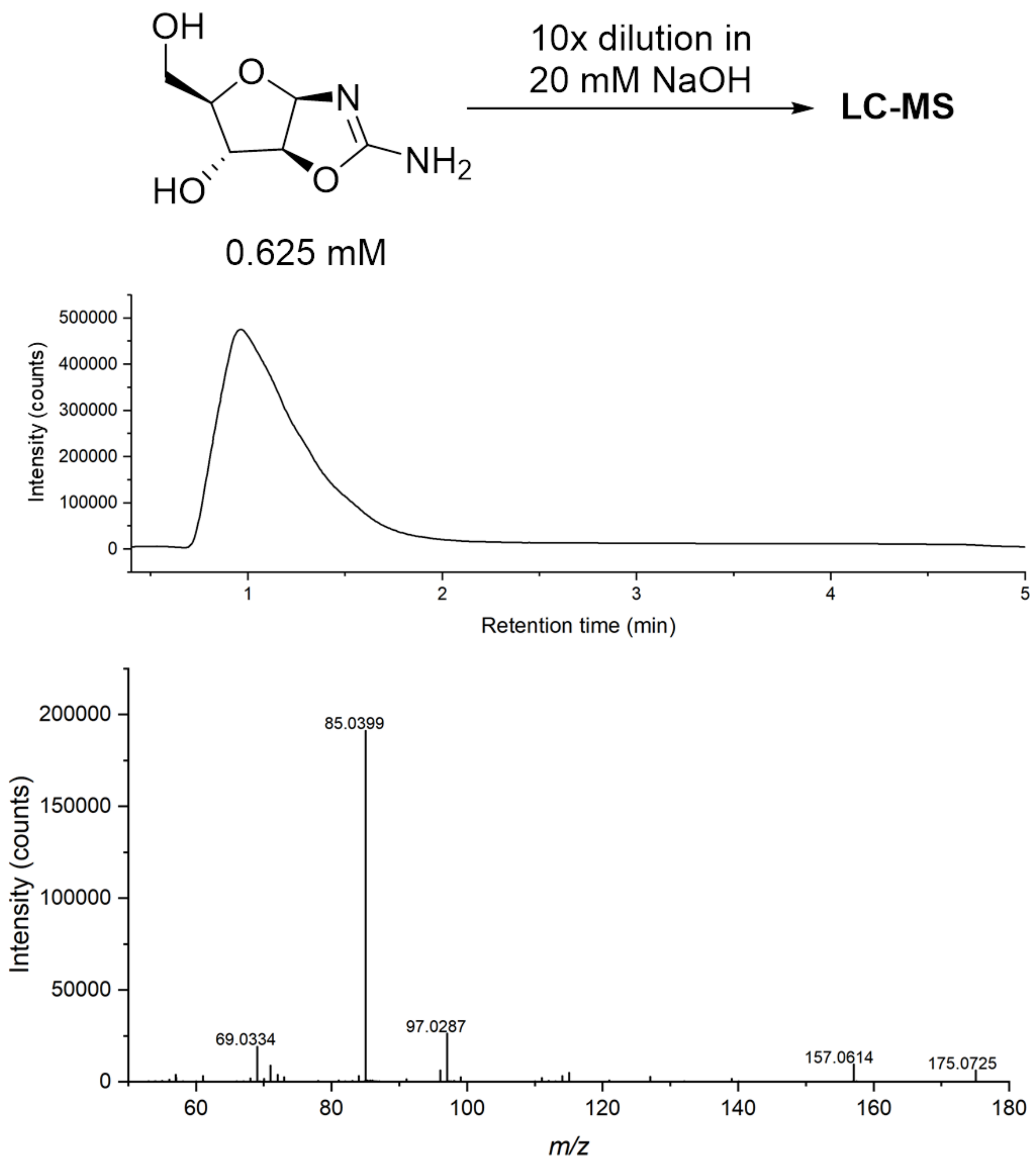

Figure S6. Arabinose aminooxazoline standard determined by LC-MS/MS analysis. Top) Procedure of arabinose aminooxazoline LC-MS/MS analysis. The standard was diluted in 20 mM NaOH to mimic the NaOH concentration of formose reaction samples after dilution. Middle) Total ion chromatogram of *m/z* 175.0719 MS/MS. Bottom) MS/MS spectrum of arabinose aminooxazoline at RT 1.023 min.

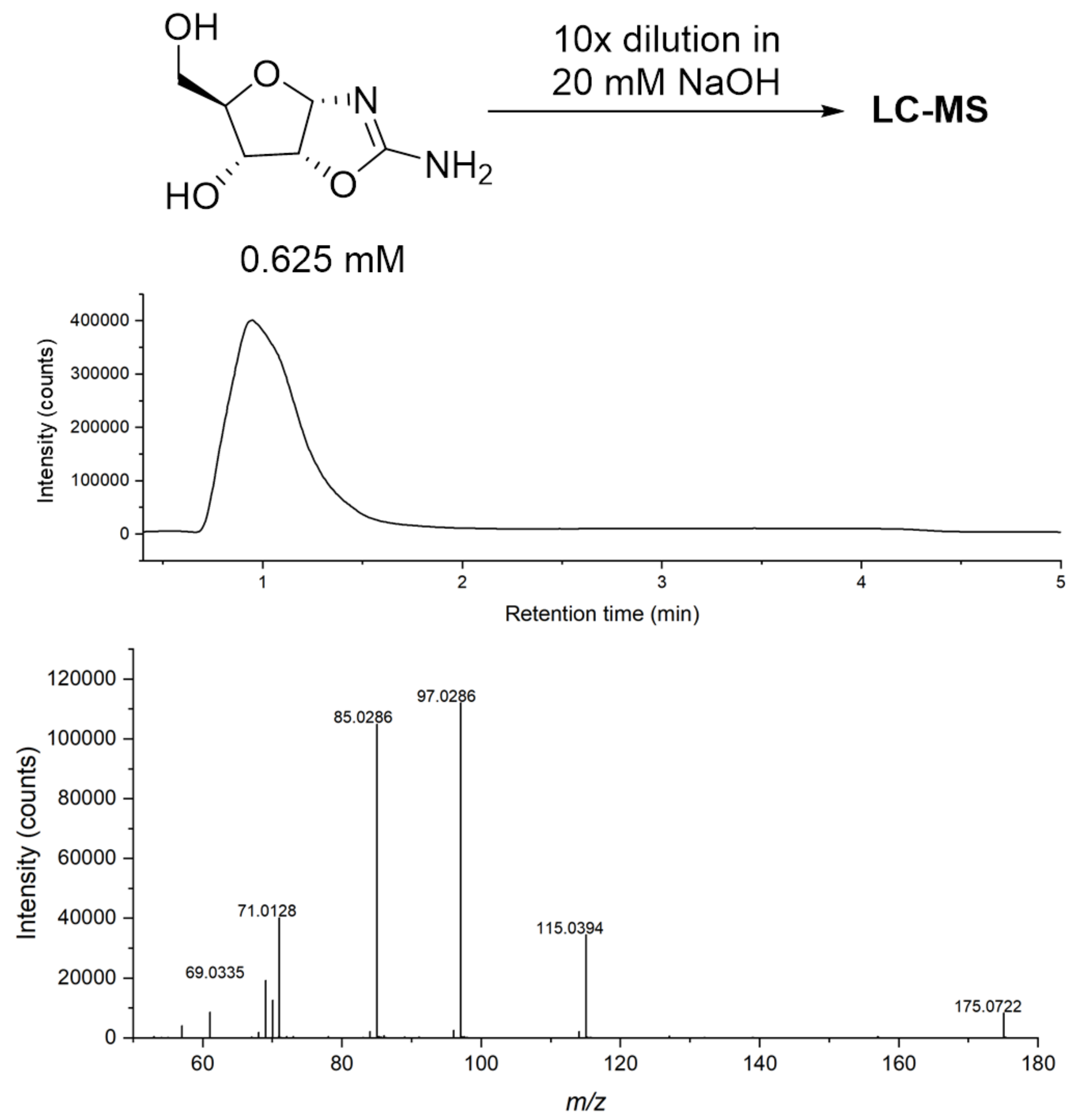

Figure S7. Ribose aminooxazoline standard determined by LC-MS/MS analysis. Top) Procedure of ribose aminooxazoline LC-MS/MS analysis. The standard was diluted in 20 mM NaOH to mimic the NaOH concentration of formose reaction samples after dilution. Middle) Total ion chromatogram of *m/z* 175.0719 MS/MS. Bottom) MS/MS spectrum of ribose aminooxazoline at RT 1.023 min.

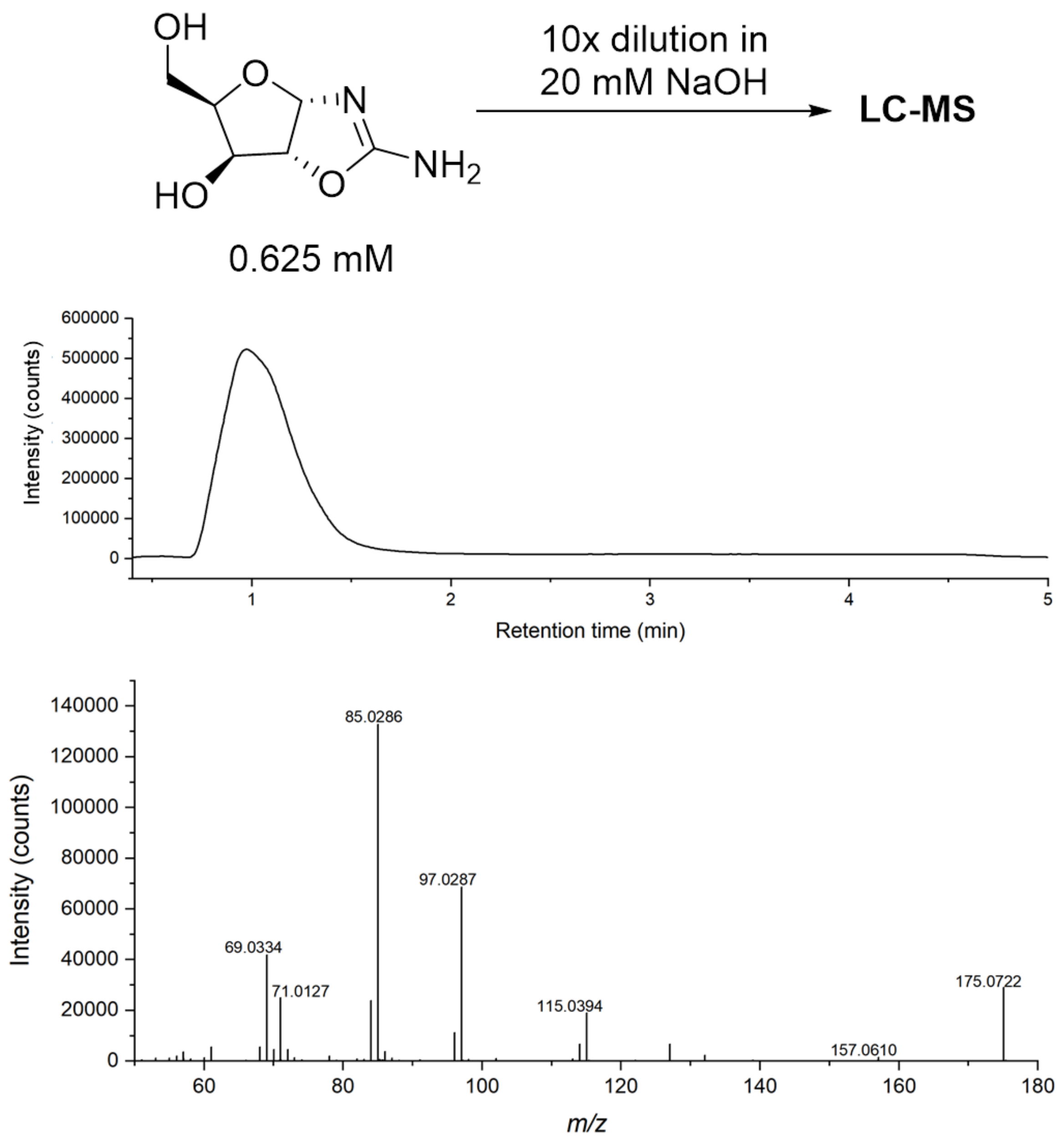

Figure S8. Xylose aminooxazoline standard determined by LC-MS/MS analysis. Top) Procedure of xylose aminooxazoline LC-MS/MS analysis. The standard was diluted in 20 mM NaOH to mimic the NaOH concentration of formose reaction samples after dilution. Middle) Total ion chromatogram of *m/z* 175.0719 MS/MS. Bottom) MS/MS spectrum of xylose aminooxazoline at RT 1.023 min.

6. Time Course Experiments of Formose Reaction with 0, 7 and 9 mM Cyanamide

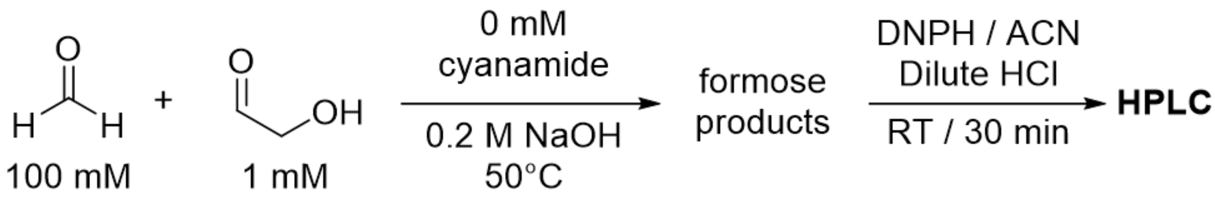

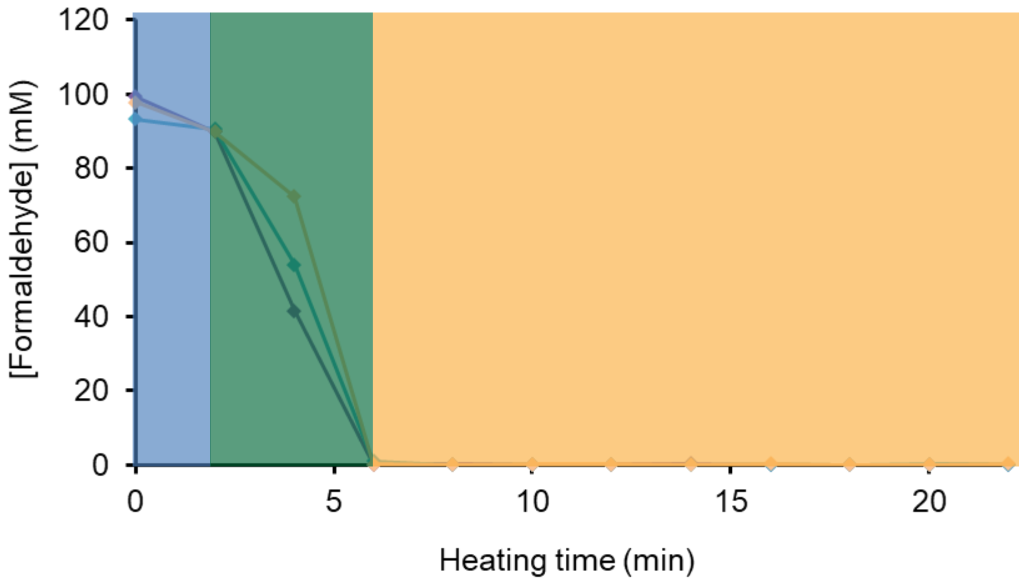

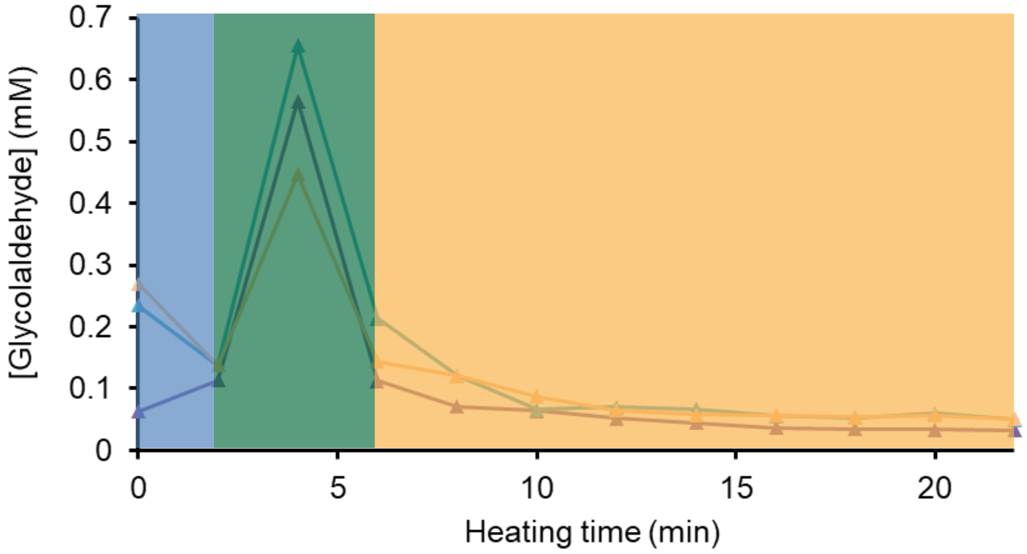

Figure S9. Concentrations of formaldehyde and glycolaldehyde over time in triplicates of the formose reaction without (0 mM) cyanamide. Concentrations of formaldehyde (middle) and glycolaldehyde (bottom) were determined through derivatisation with DNPH followed by analysis with HPLC (see Figure S12 for extracted chromatograms at 360 nm). The regions are colour-coded based on their kinetic phase–lag phase (blue), exponential (green), and degradation (yellow). Their concentrations were calculated based on their respective standard curves (see Figures S2 and S3). At 0 mM initial cyanamide, the exponential phase started almost immediately after heating.

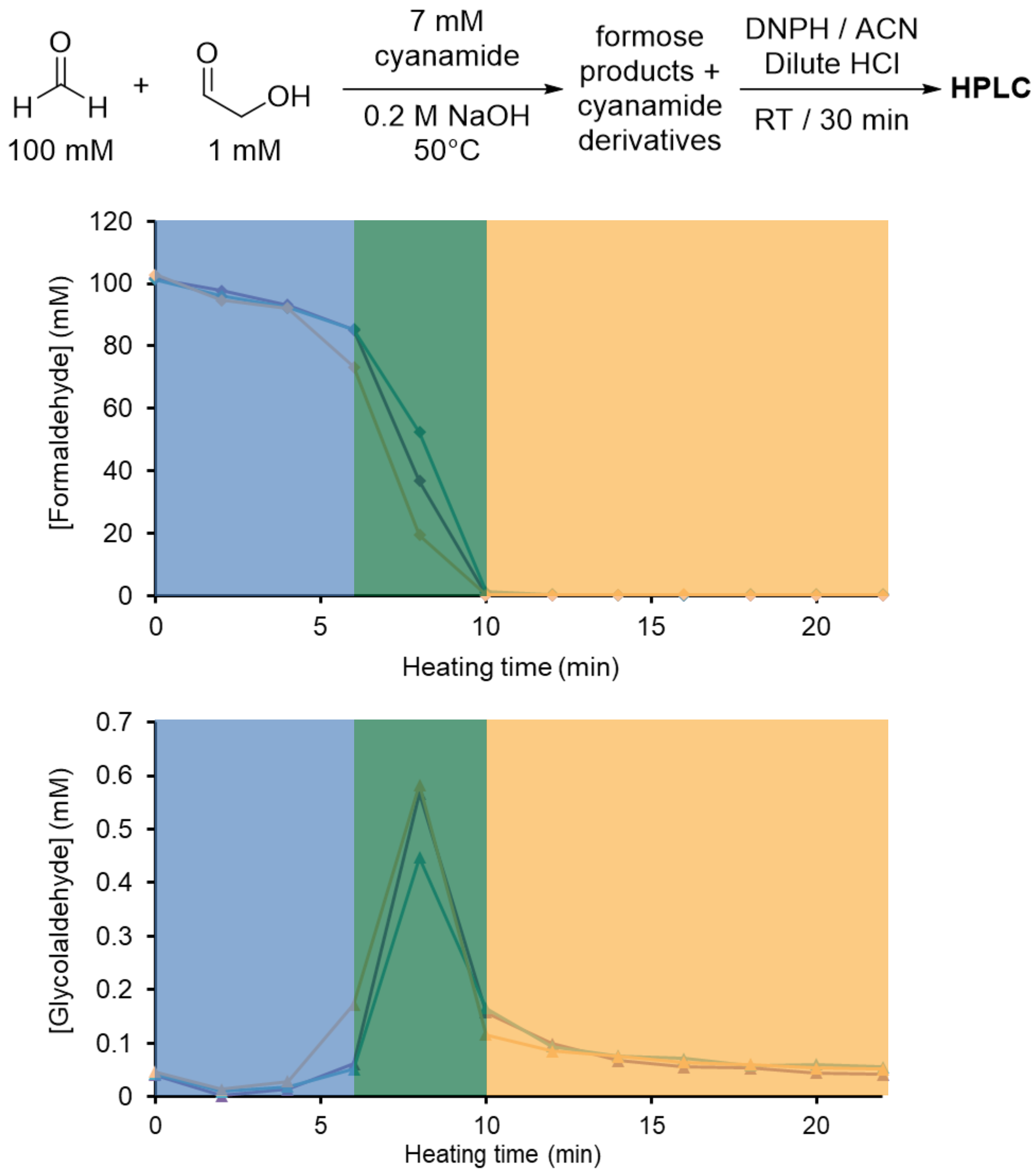

Figure S10. Concentrations of formaldehyde and glycolaldehyde over time in triplicates of the formose reaction containing 7 mM cyanamide. Concentrations of formaldehyde (middle) and glycolaldehyde (bottom) were determined through derivatisation with DNPH followed by analysis with HPLC (see Figure S13 for extracted chromatograms at 360 nm). The regions are colour-coded based on their kinetic phase–lag phase (blue), exponential (green), and degradation (yellow). Their concentrations were calculated based on their respective standard curves (see Figures S2 and S3). At 7 mM initial cyanamide, the exponential phase was delayed to ~6 minutes after heating.

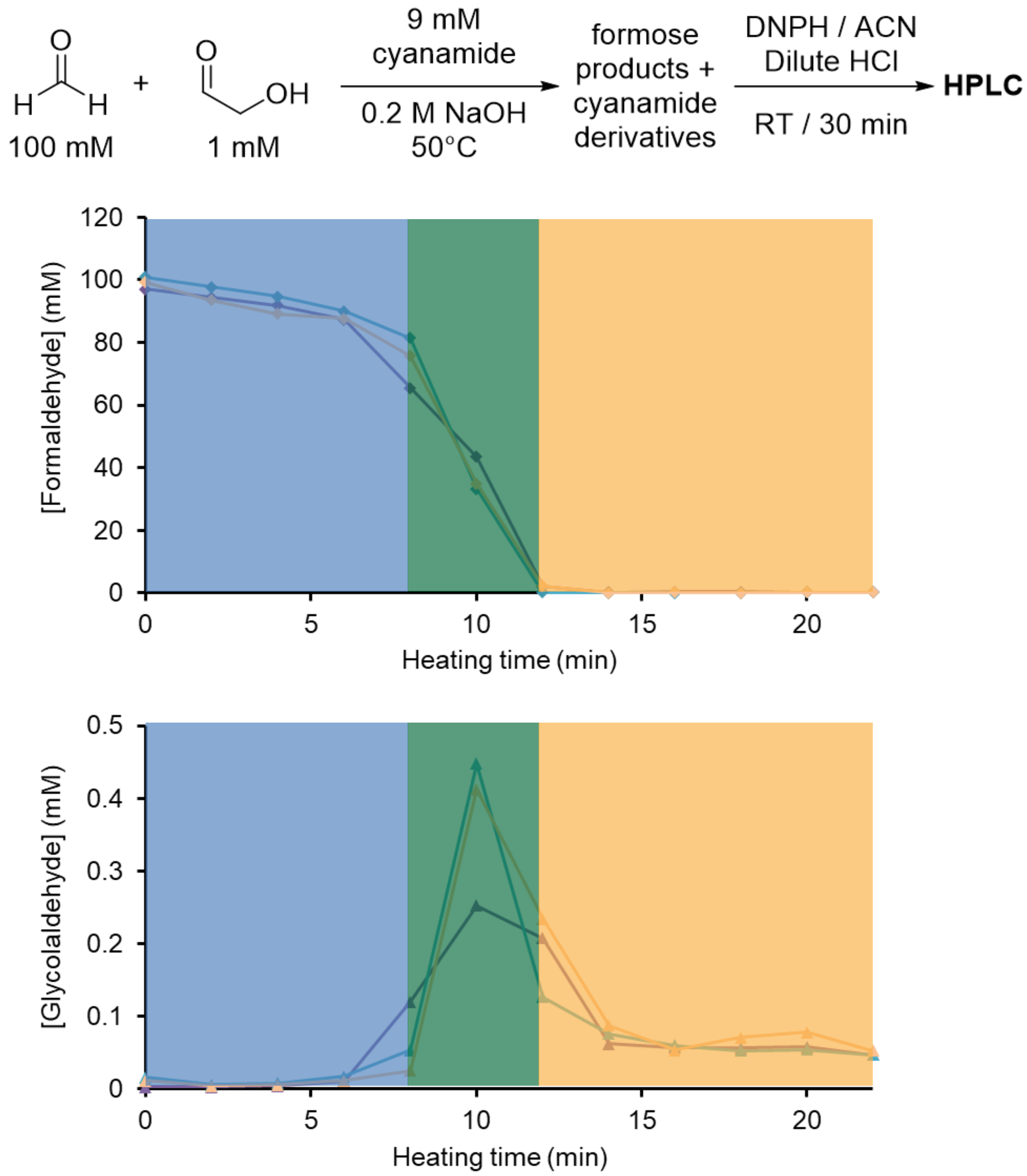

Figure S11. Concentrations of formaldehyde and glycolaldehyde over time in triplicates of the formose reaction containing 9 mM cyanamide. Concentrations of formaldehyde (middle) and glycolaldehyde (bottom) were determined through derivatisation with DNPH followed by analysis with HPLC (see Figure S14 for extracted chromatograms at 360 nm). The regions are colour-coded based on their kinetic phase–lag phase (blue), exponential (green), and degradation (yellow). Their concentrations were calculated based on their respective standard curves (see Figures S2 and S3). At 9 mM initial cyanamide, the exponential phase was further delayed to ~8 minutes after heating.

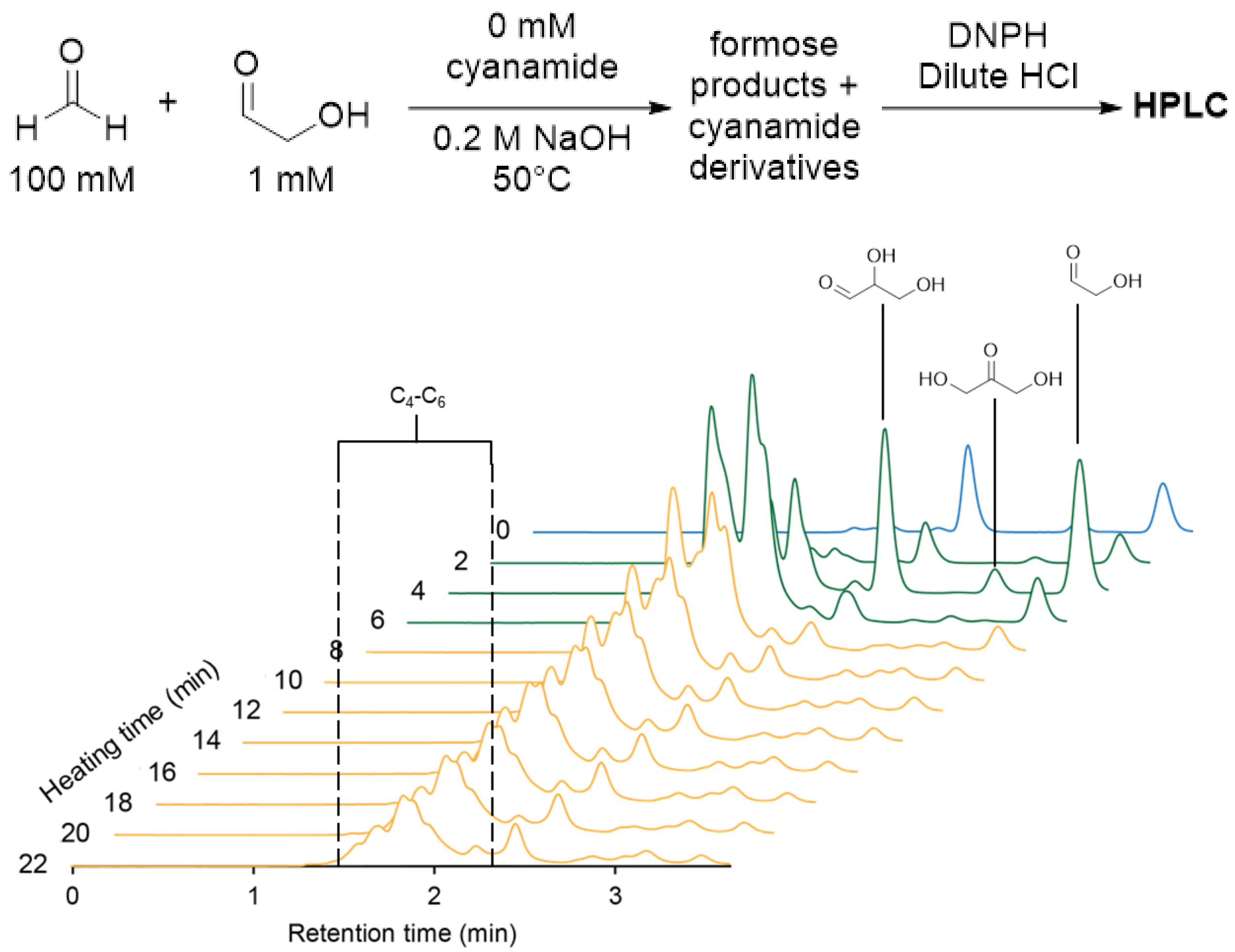

Figure S12. Extracted HPLC chromatograms at 360 nm absorbance of formose reaction timepoints containing 0 mM initial cyanamide. Formose reaction timepoints were derivatised with DNPH followed by HPLC analysis. The chromatograms are colour-coded based on which phase of the formose reaction they belong to. Blue chromatograms represent the lag phase, green the exponential phase, and yellow the degradation phase following the yellowing point. Without initial cyanamide, the formose reaction quickly reached the exponential phase, producing noticeable amounts of C_3–C_6 sugars, evident in the increased peak areas. After the yellowing point (~6 min), the peaks gradually decreased in intensity. All chromatographic peaks shown display an absorption spectrum consistent with DNPH derivatisation.

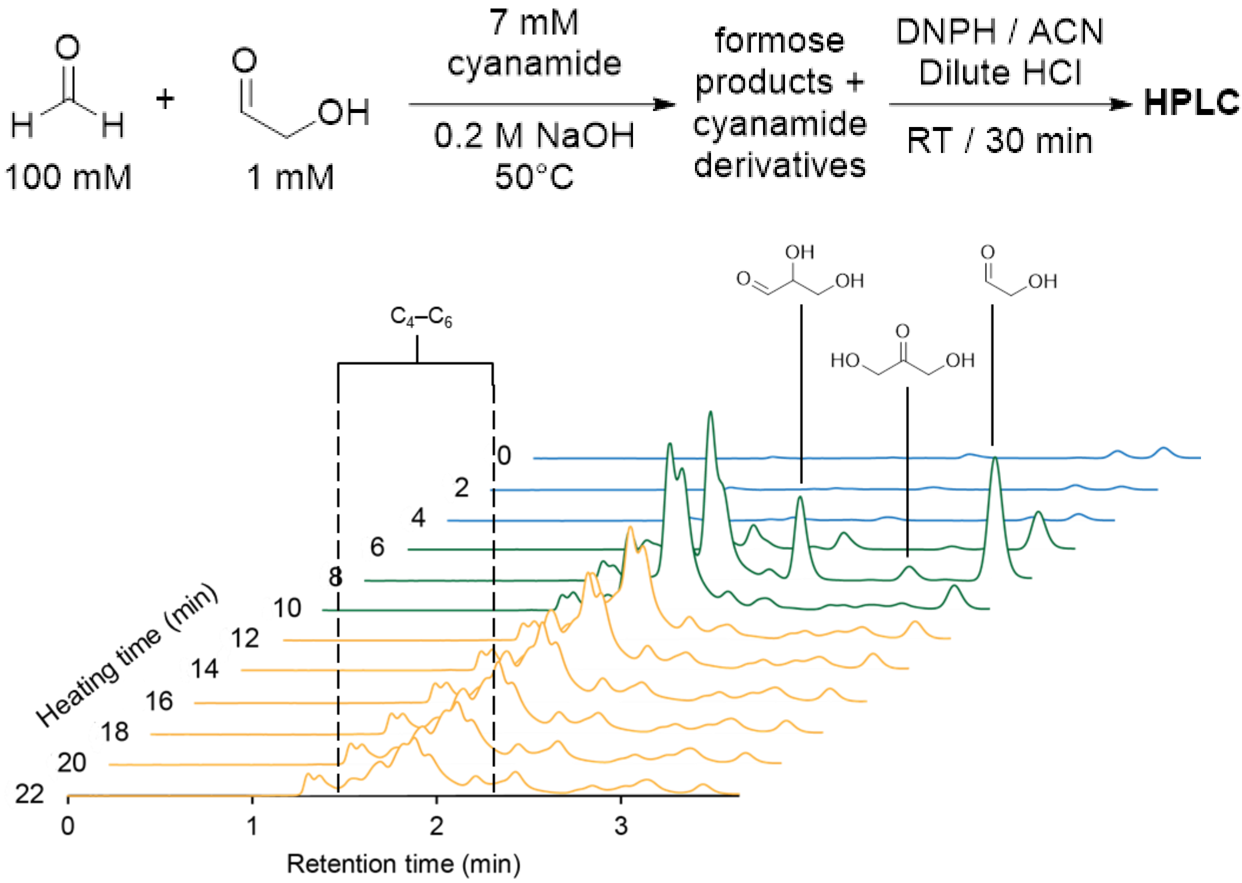

Figure S13. Extracted HPLC chromatograms at 360 nm absorbance of formose reaction timepoints containing 7 mM initial cyanamide. Formose reaction timepoints were derivatised with DNPH followed by HPLC analysis. The chromatograms are colour coded based on which phase of the formose reaction they belong to. Blue chromatograms represent the lag phase, green the exponential phase, and yellow the degradation phase following the yellowing point. With 7 mM initial cyanamide, the lag phase of the formose reaction was extended by ~4 minutes. The amounts of C_2–C_6 sugars produced during the exponential phase were noticeably lower than the formose reaction without cyanamide (Figure S12). After the yellowing point (~10 min), the peaks gradually decrease in intensity. All chromatographic peaks shown display an absorption spectrum consistent with DNPH derivatisation.

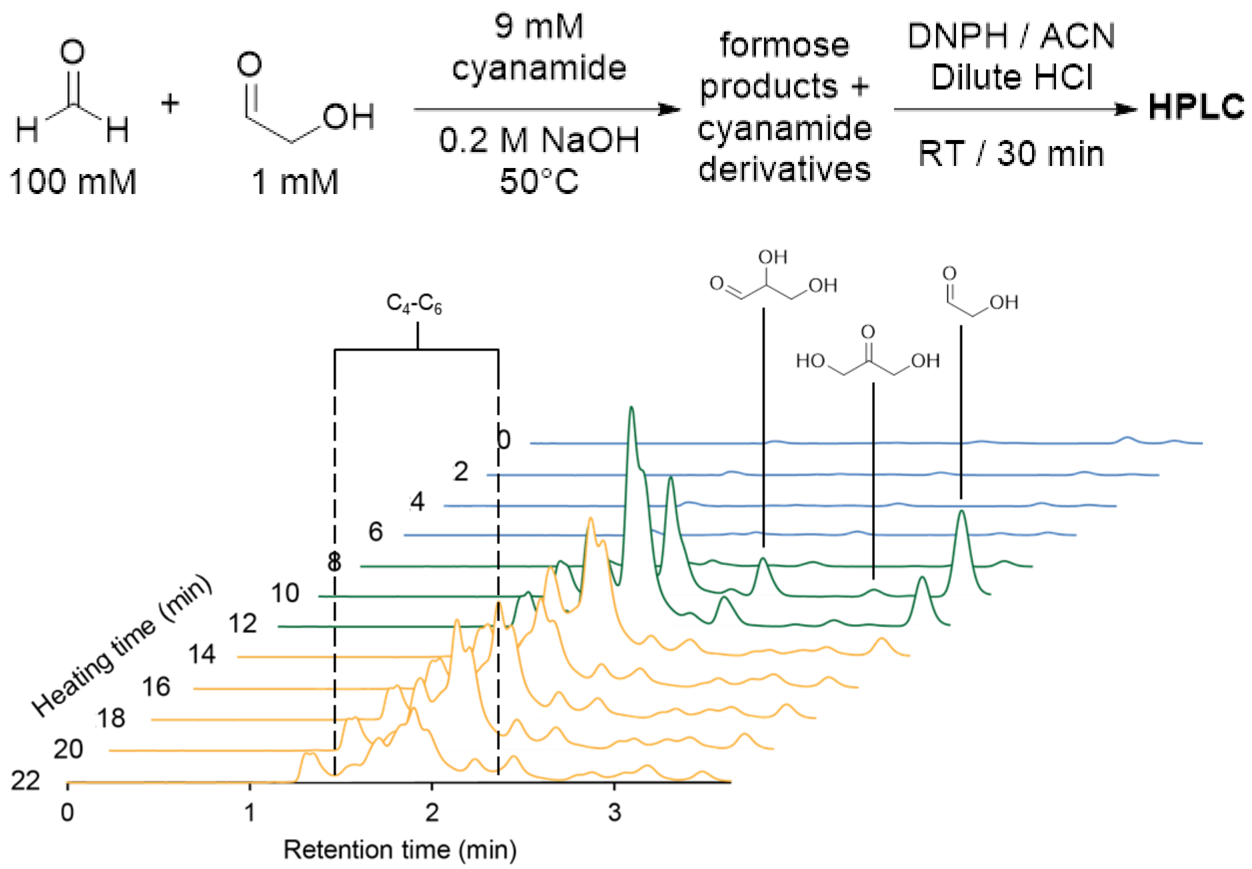

Figure S14. Extracted HPLC chromatograms at 360 nm absorbance of formose reaction timepoints containing 9 mM initial cyanamide. Formose reaction timepoints were derivatised with DNPH followed by HPLC analysis. The chromatograms are colour coded based on which phase of the formose reaction they belong to. Blue chromatograms represent the lag phase, green the exponential phase, and yellow the degradation phase following the yellowing point. With 9 mM initial cyanamide, the lag phase of the formose reaction was extended by ~6 minutes. The amounts of C_3-C_6 sugars produced during the exponential phase were noticeably lower than the formose reaction without cyanamide (Figure S12) but comparable to the reaction with 7 mM cyanamide (Figure S13). After the yellowing point (~12 min), the peaks gradually decrease in intensity. All chromatographic peaks shown display an absorption spectrum consistent with DNPH derivatisation.

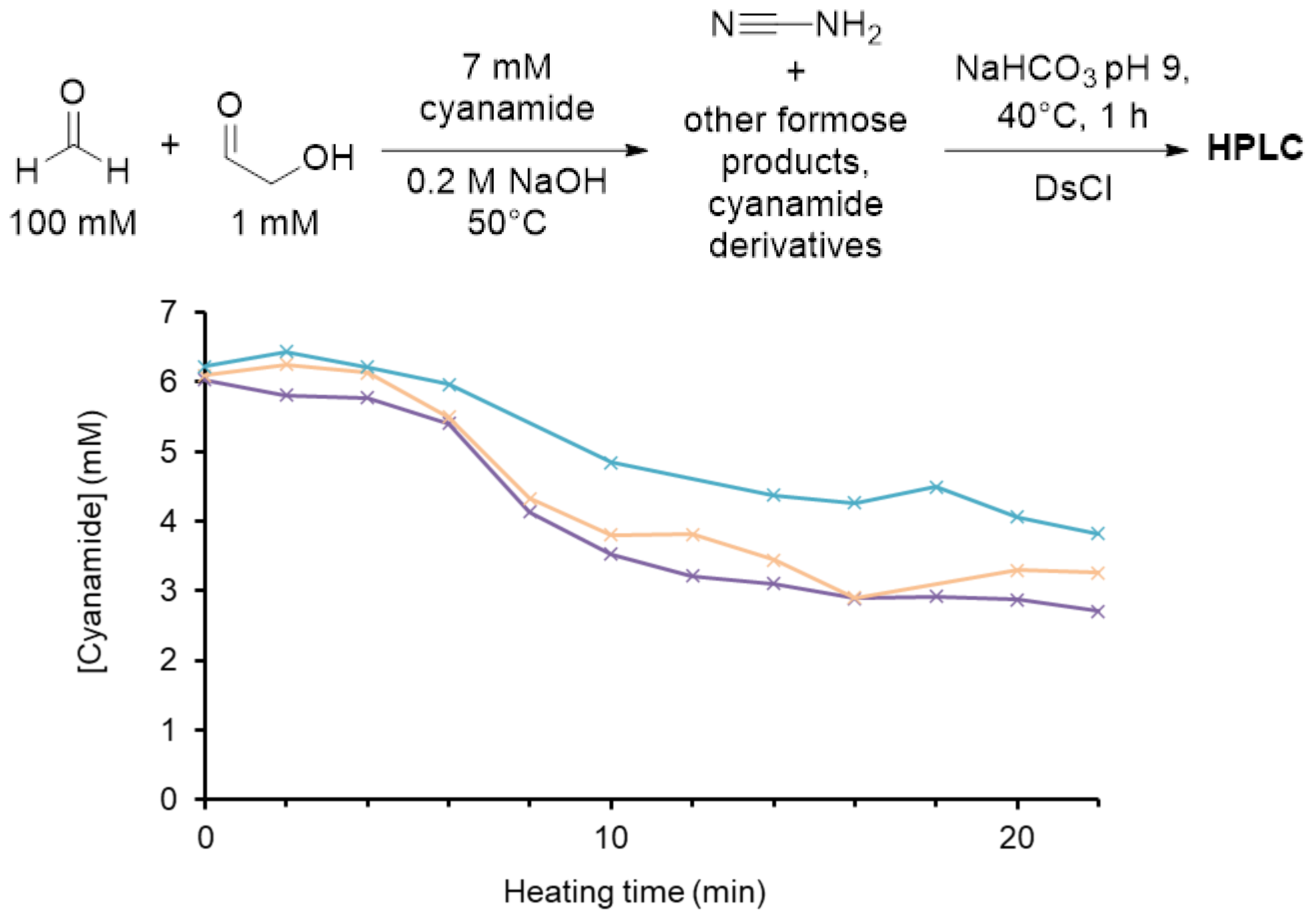

Figure S15. Concentration of cyanamide over time in triplicates of the formose reaction containing 7 mM initial cyanamide. Concentration of cyanamide was determined through derivatisation with dansyl chloride followed by analysis with HPLC at 350 nm (see Figure S16 for extracted chromatograms). Concentration was calculated based on the respective standard curve (see Figure S4). Concentration of cyanamide prior to heating was determined to be ~6 mM instead of 7 mM which could be accounted for by its reaction with glycolaldehyde and other formose products that start to form immediately after mixing. The consumption of cyanamide was observed to reflect the kinetic phase of the formose reaction. Cyanamide concentration started decreasing significantly at the onset of the exponential phase (~6 minutes). Cyanamide consumption slowed down around ~10–12 minutes (the yellowing point) and concentration stabilised afterwards.

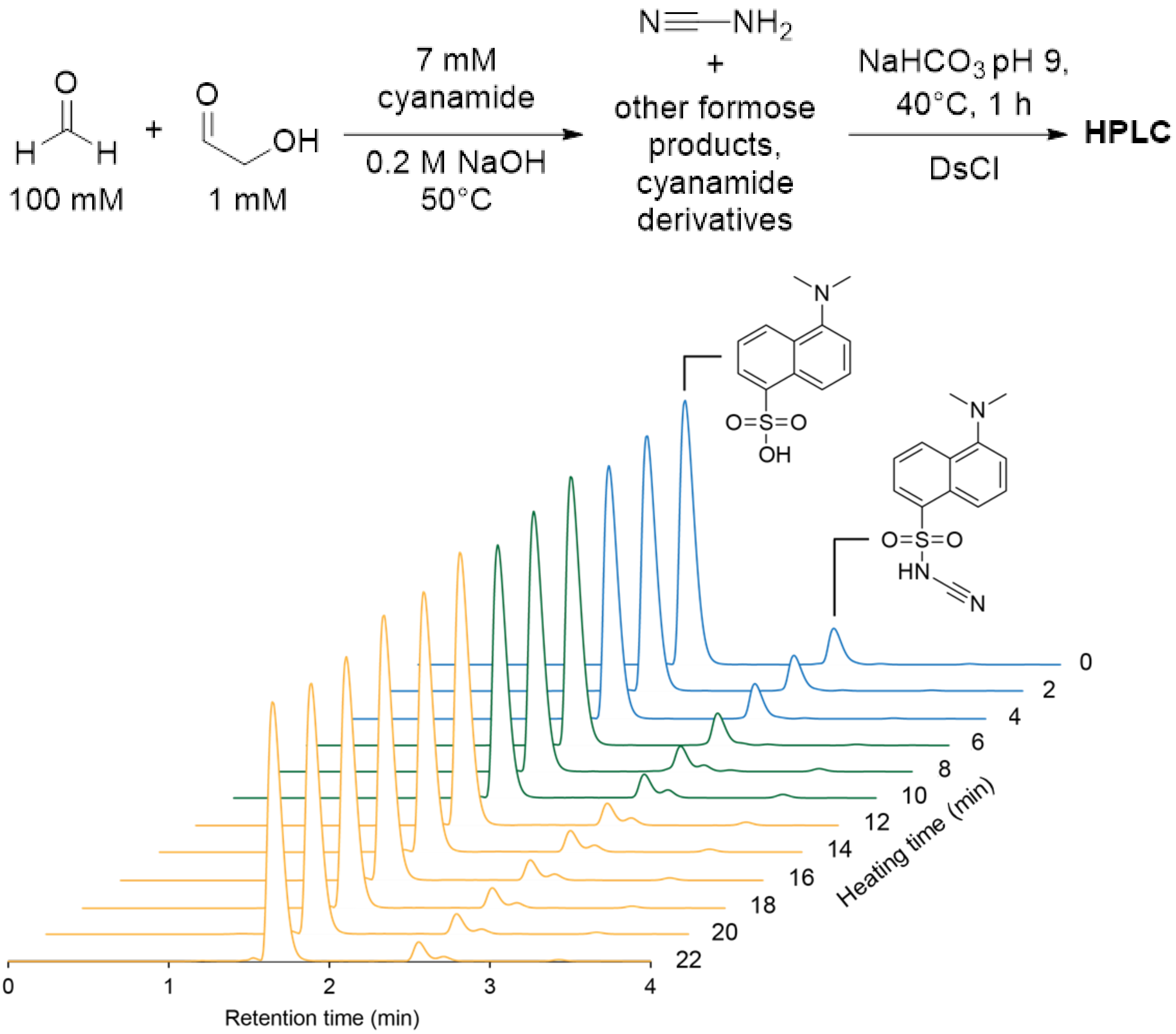

Figure S16. Extracted HPLC chromatograms at 350 nm absorbance of formose reaction timepoints containing 7 mM initial cyanamide. Formose reaction timepoints were derivatised with dansyl chloride followed by HPLC analysis. The chromatograms are colour coded based on which phase of the formose reaction they belong to. Blue chromatograms represent the lag phase, green the exponential phase, and yellow the degradation phase. Integration of the cyanamide peak revealed that cyanamide consumption reflected the kinetic phase of the formose reaction.

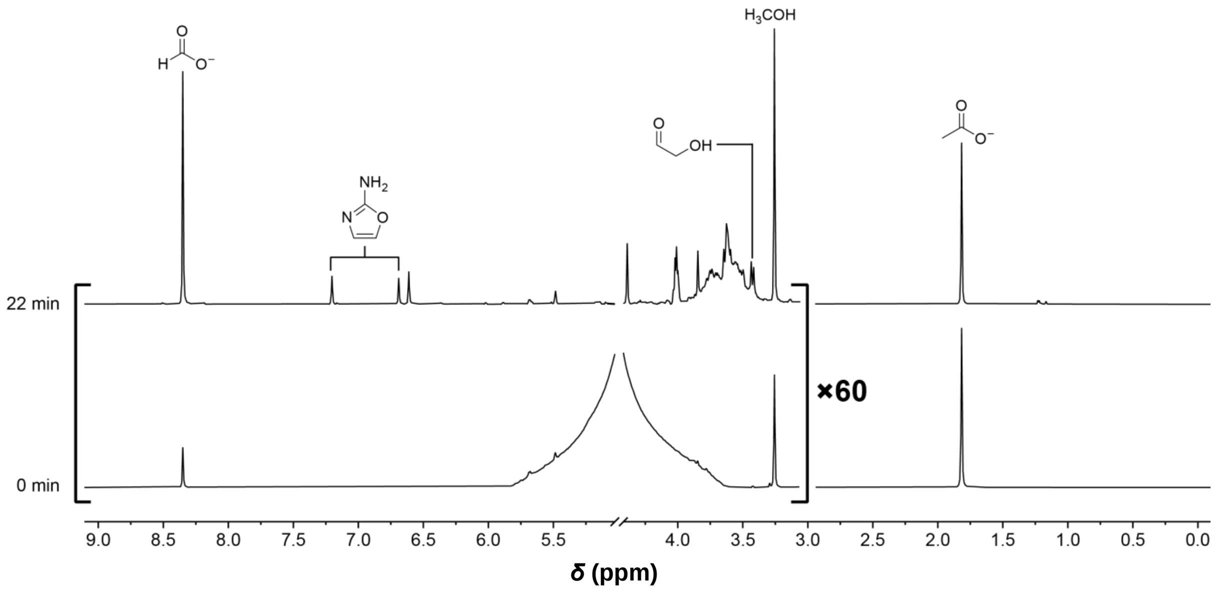

Figure S17. ^{1}H NMR spectra of formose reaction containing 7 mM initial cyanamide before and after 22 minutes of heating at 50°C. The presence of 2-NH2Ox, glycolaldehyde, acetate, and methanol were confirmed via addition of standards (see Figures S18–19) and formate via comparison with literature.

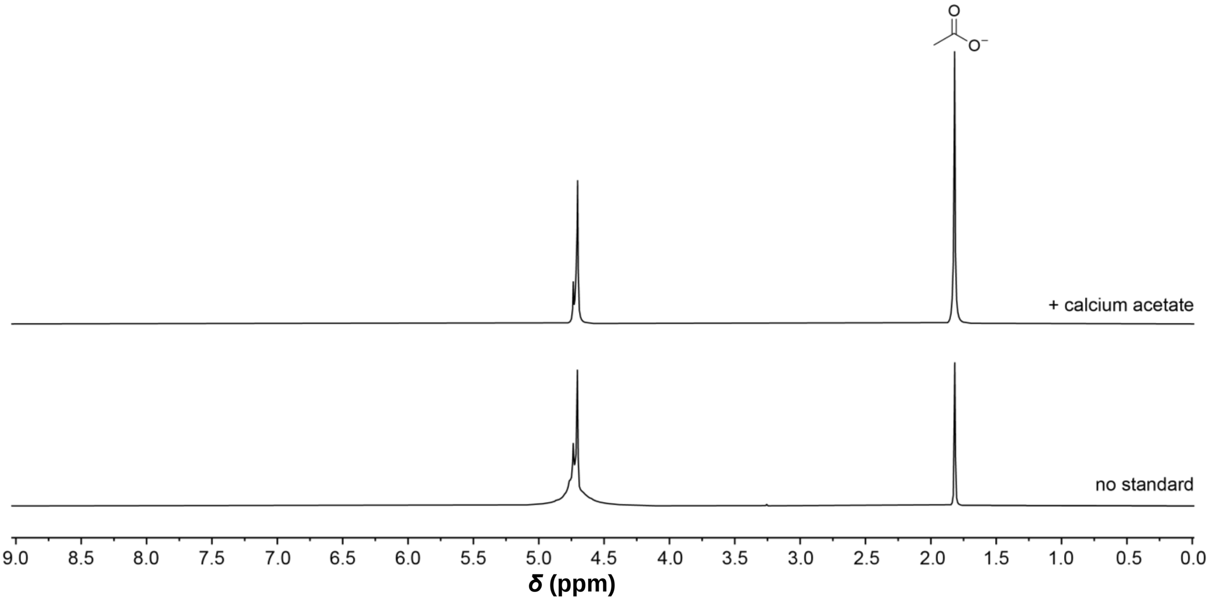

Figure S18. ^{1}H NMR spectra of formose reaction containing 7 mM initial cyanamide before heating with and without calcium acetate standard.

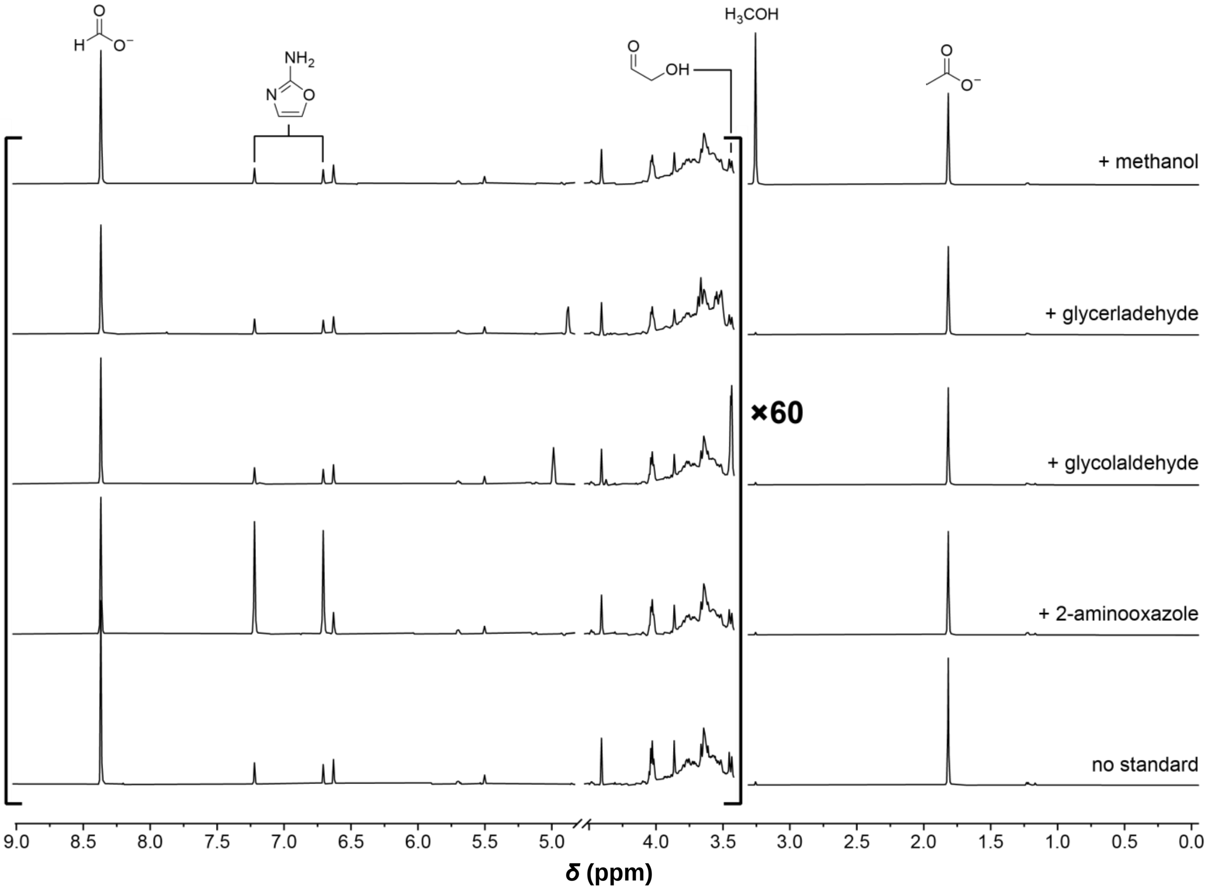

Figure S19. ^1H NMR spectra of formose reaction containing 7 mM initial cyanamide after 22 minutes of heating at 50 °C with and without standards (2-NH_2Ox, glycolaldehyde, glyceraldehyde, and methanol).

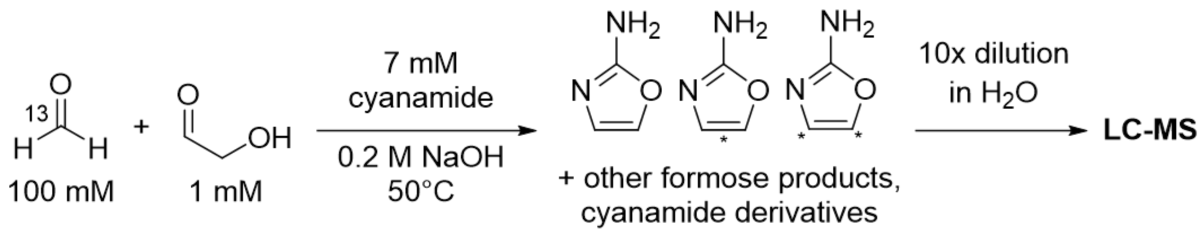

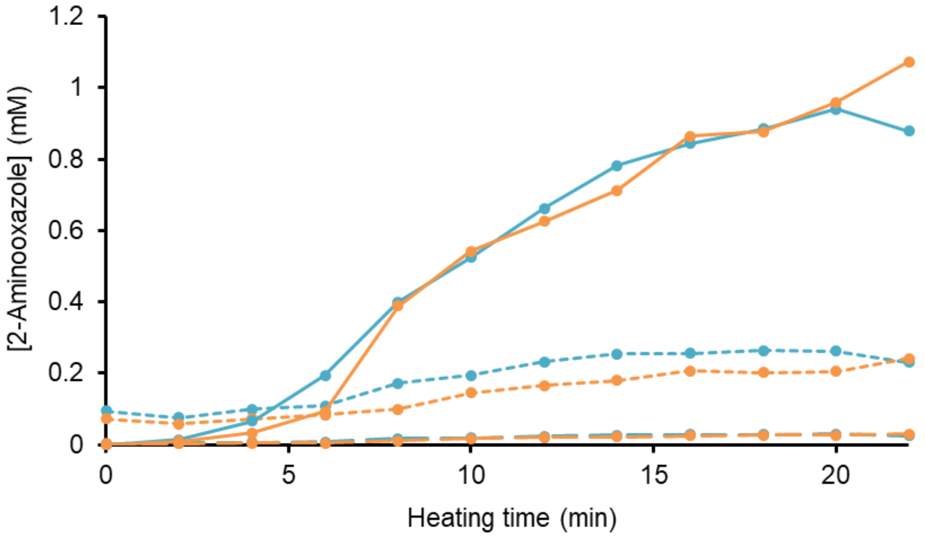

Figure S20. Concentration of $C_3H_4N_2O$ (2-NH_2Ox)) with 0, 1, and 2 ^{13}C isotopes over time (in two replicates) of the formose reaction containing 7 mM initial cyanamide and 100 mM $^{13}CH_2O$. 2-NH_2Ox concentrations were calculated using the standard curve in Figure S5. Before heating, only unlabelled 2-NH_2Ox (m/z_{obs} 85.0396) was observed. However, heating initiated the production of ^{13}C-labelled glycolaldehyde (and other formose intermediates) leading to the formation of doubly-^{13}C-labelled 2-NH_2Ox (m/z_{obs} 87.0456). By the end of the observation window, the doubly-^{13}C-labelled species has taken over the unlabelled species in concentration. Trace amounts of singly-^{13}C-labelled 2-NH_2Ox (m/z_{obs} 86.0422) were also observed.

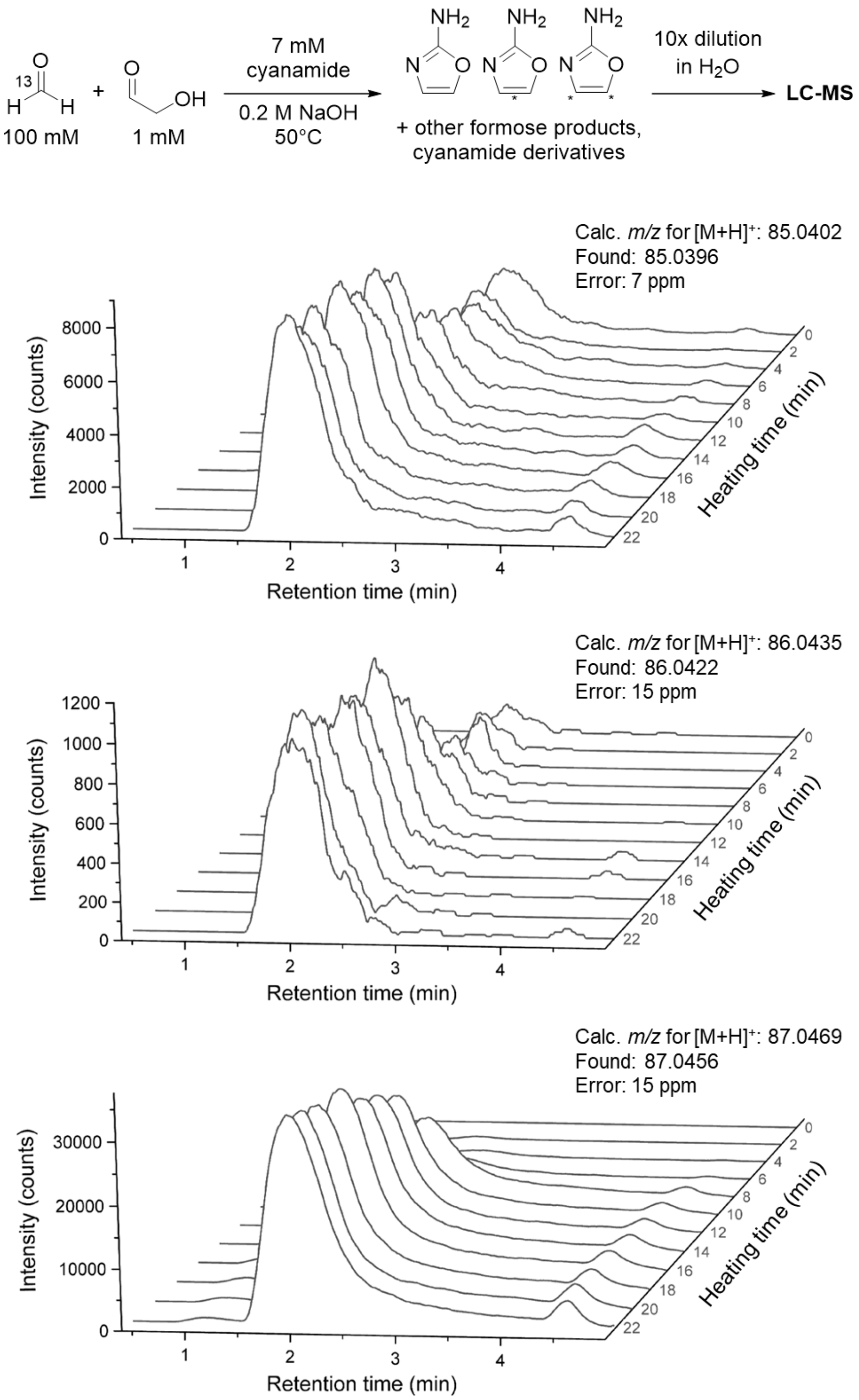

Figure S21. Extracted ion chromatograms (EICs) of $C_3H_4N_2O$ (2-NH_2Ox) with 0, 1, and 2 ^{13}C isotopes in formose reaction timepoints containing 7 mM initial cyanamide and 100 mM $^{13}CH_2O$. A mass range of +/– 10 ppm, based on found mass, was applied for each EIC. Non-labelled $C_3H_4N_2O$ (m/z_{obs} 85.0396) increased only slightly from the start to the end of the observation window. In contrast, the concentration of doubly-^{13}C-labelled $C_3H_4N_2O$ (m/z_{obs} 87.0456) started at 0 and increased to become the dominant species, demonstrating the majority of $C_3H_4N_2O$ was produced from formose-derived glycolaldehyde. Trace amounts of singly-^{13}C-labelled $C_3H_4N_2O$ (m/z_{obs} 86.0422) were also detected.

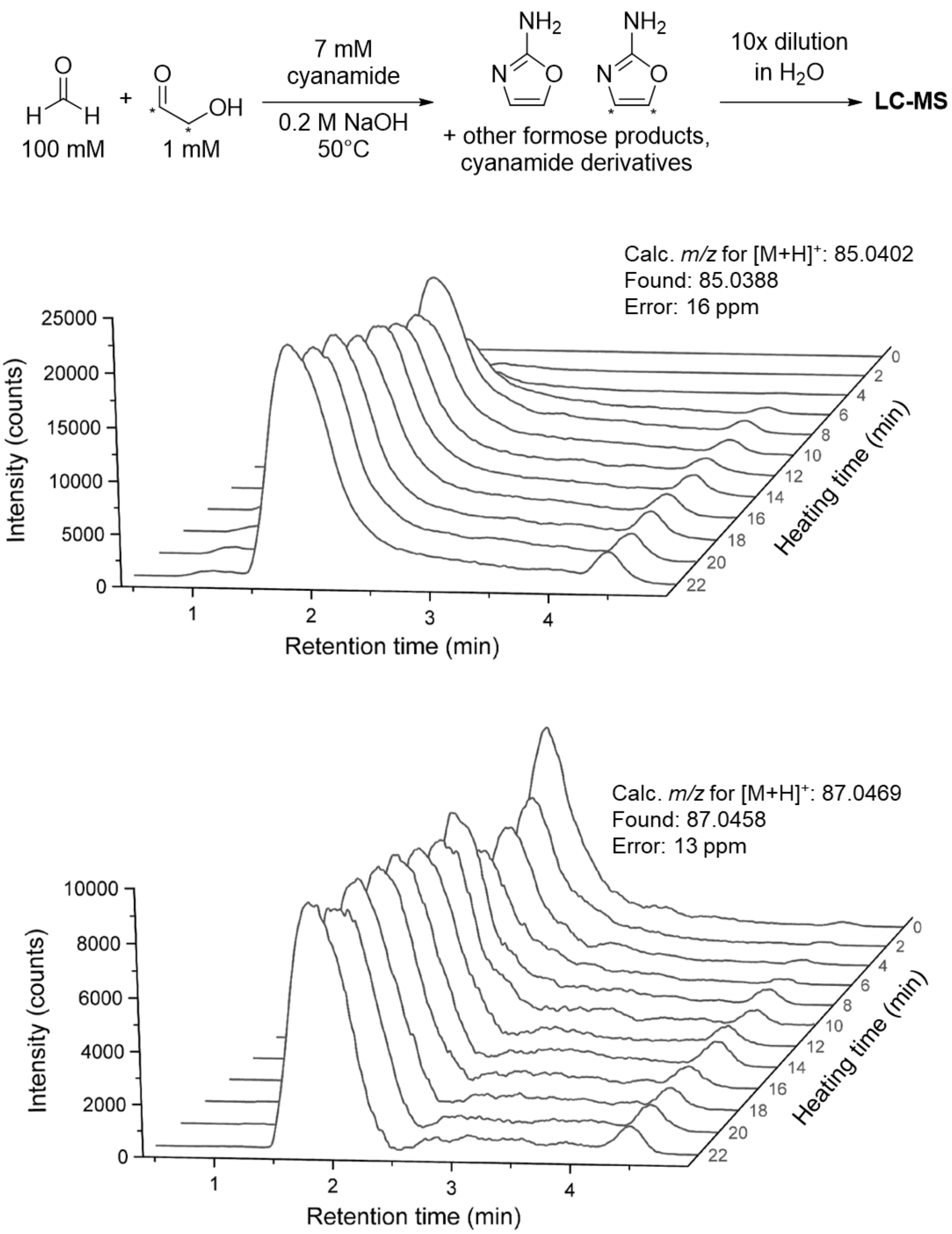

Figure S22. Extracted ion chromatograms of $C_3H_4N_2O$ (2-NH_2Ox) in formose reaction timepoints containing 7 mM initial cyanamide and 1 mM initial glycolaldehyde-1,2-$^{13}C_2$. A mass range of +/− 10 ppm, based on found mass, was applied for each EIC. The concentration of doubly-^{13}C-labelled 2-NH_2Ox (m/z_{obs} 87.0458), which formed predominantly at the start, remained relatively constant. Unlabelled 2-NH_2Ox (m/z_{obs} 85.0388) started at 0 and increased significantly at the onset of the exponential phase. After the yellowing point, the rate of increase slowed down.

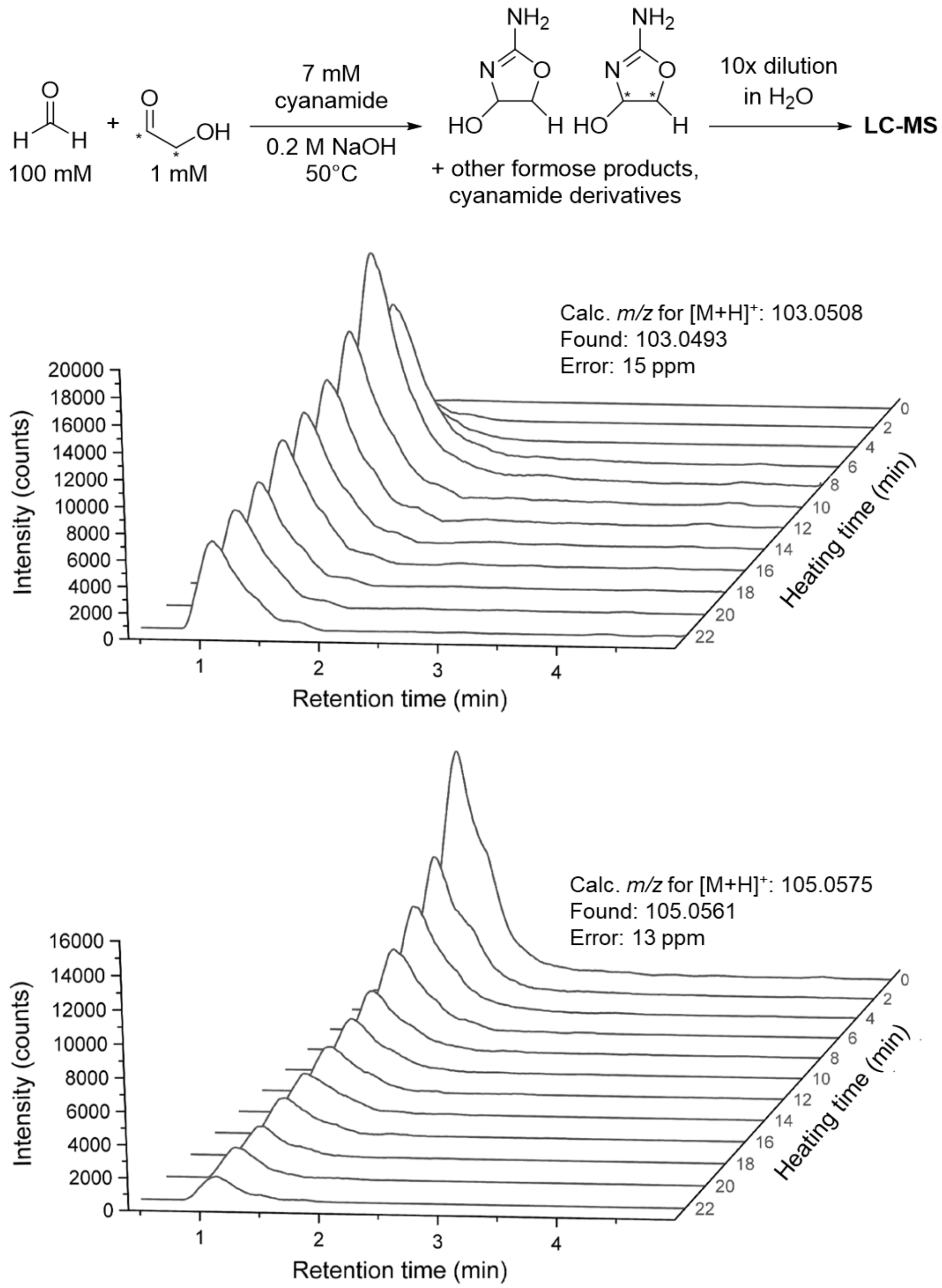

Figure S23. Extracted ion chromatograms of $C_3H_6N_2O_2$ (interpreted as the 2-NH_2Ox hydrate) in formose reaction timepoints containing 7 mM cyanamide and 1 mM initial glycolaldehyde-1,2-$^{13}C_2$. A mass range of +/− 10 ppm, based on found mass, was applied for each EIC. The concentration of the doubly-^{13}C-labelled species (m/z_{obs} 105.0561), which formed predominantly at the start, decreased continuously throughout the experiment. The unlabelled species (m/z_{obs} 103.0493) started at 0, reached maximum concentration during the exponential phase (~8 min). For the rest of the experiment, the unlabelled species decreased, which could explain the increase in 2-NH_2Ox, which is more stable due to aromaticity.

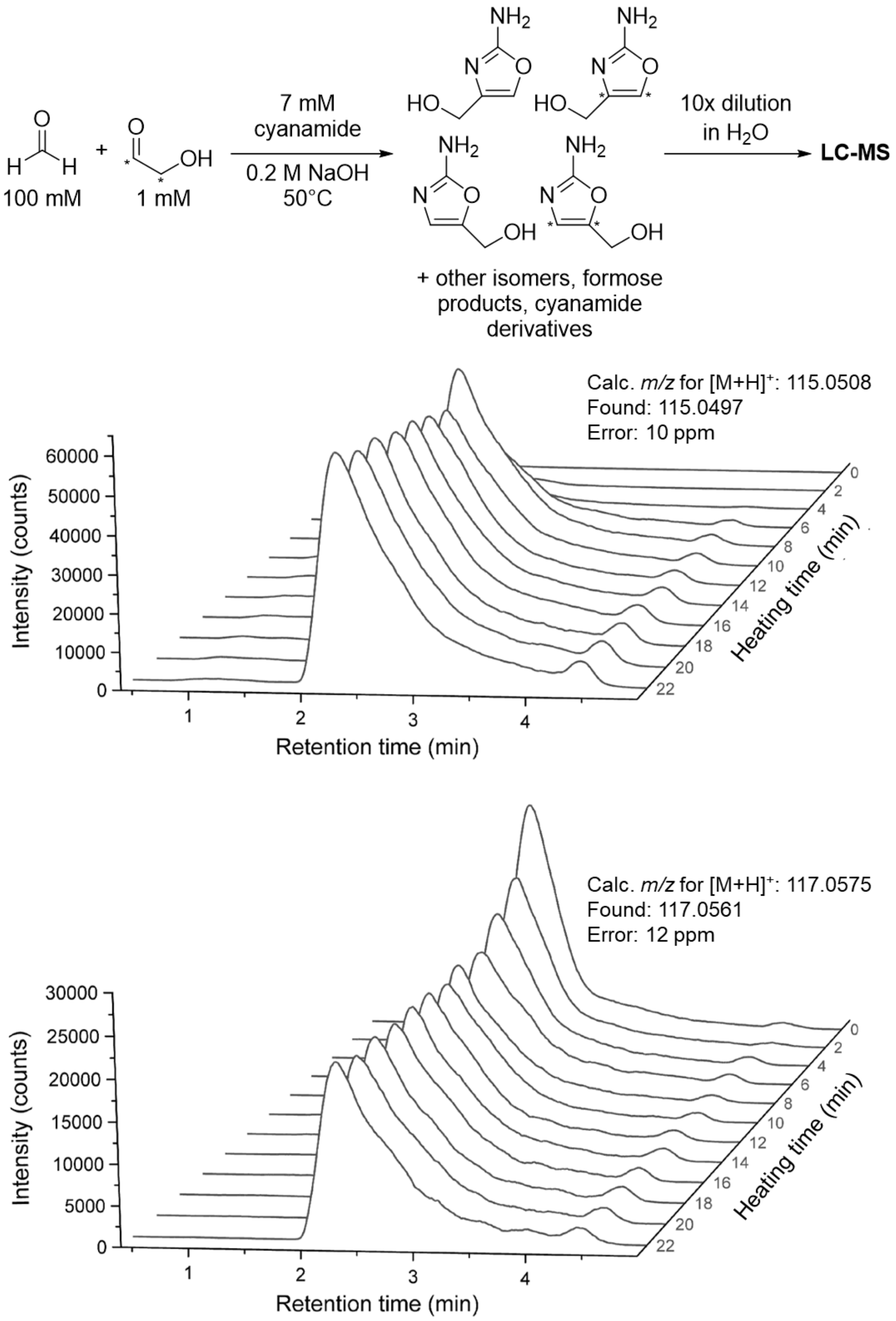

Figure S24. Extracted ion chromatograms of $C_4H_6N_2O_2$ (C_3-cyanamide adduct anhydrate) in formose reaction timepoints containing 7 mM cyanamide and 1 mM initial glycolaldehyde-1,2-$^{13}C_2$. A mass range of +/– 10 ppm, based on found mass, was applied for each EIC. The structures shown in the scheme above are proposed and consistent with the observed masses. See Table S1 for proposed structures of aminooxazoles derivatives. The concentration of the doubly-^{13}C-labelled species (m/z_{obs} 117.0561), which formed predominantly at the start, remained relatively constant. The unlabelled species (m/z_{obs} 115.0497) started at 0 and increased significantly at the onset of the exponential phase. After the yellowing point, the rate of increase slowed down.

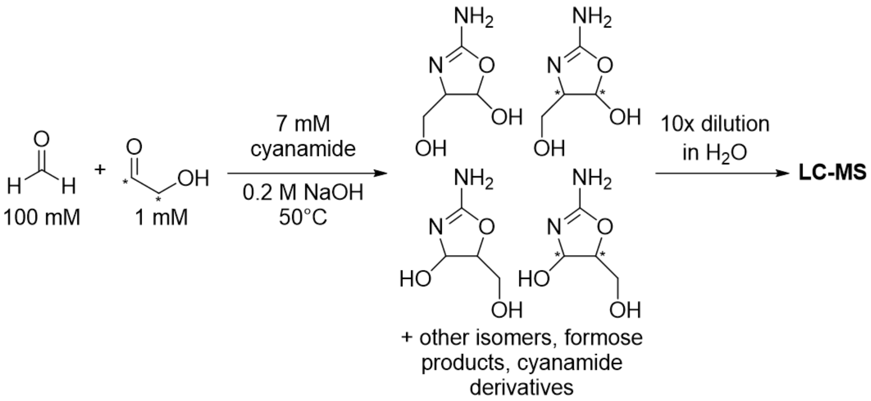

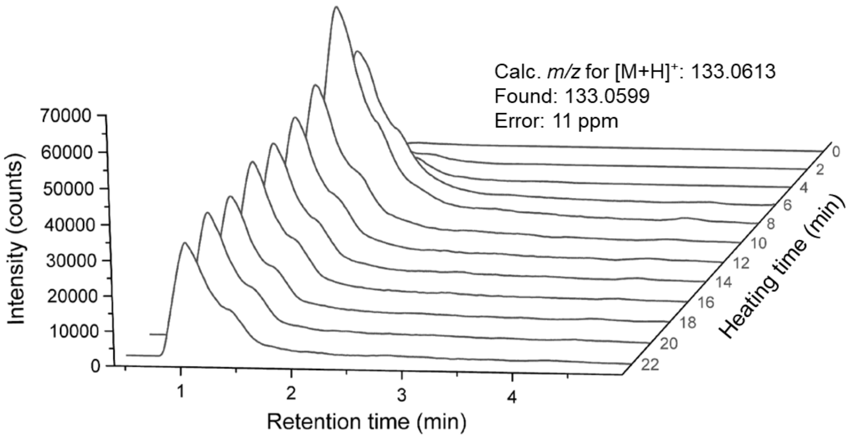

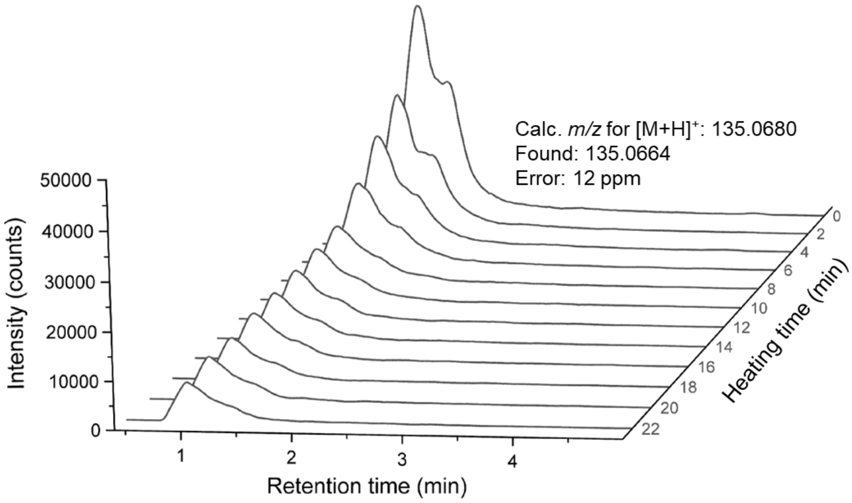

Figure S25. Extracted ion chromatograms of $C_4H_8N_2O_3$ (C_3-cyanamide adduct) in formose reaction timepoints containing 7 mM cyanamide and 1 mM initial glycolaldehyde-1,2-$^{13}C_2$. A mass range of +/– 10 ppm, based on found mass, was applied for each EIC. The structures shown in the scheme above are proposed and consistent with the observed masses. See Table S1 for proposed structures of aminooxazoles derivatives. The concentration of the doubly-^{13}C-labelled species (m/z_{obs} 135.0664), which formed predominantly at the start, decreased continuously throughout the experiment. The unlabelled species (m/z_{obs} 133.0599) started at 0, reached maximum concentration during the exponential phase (~8 min). For the rest of the experiment, the unlabelled species decreased, which could explain the increase of the anhydrate, which is more stable due to aromaticity.

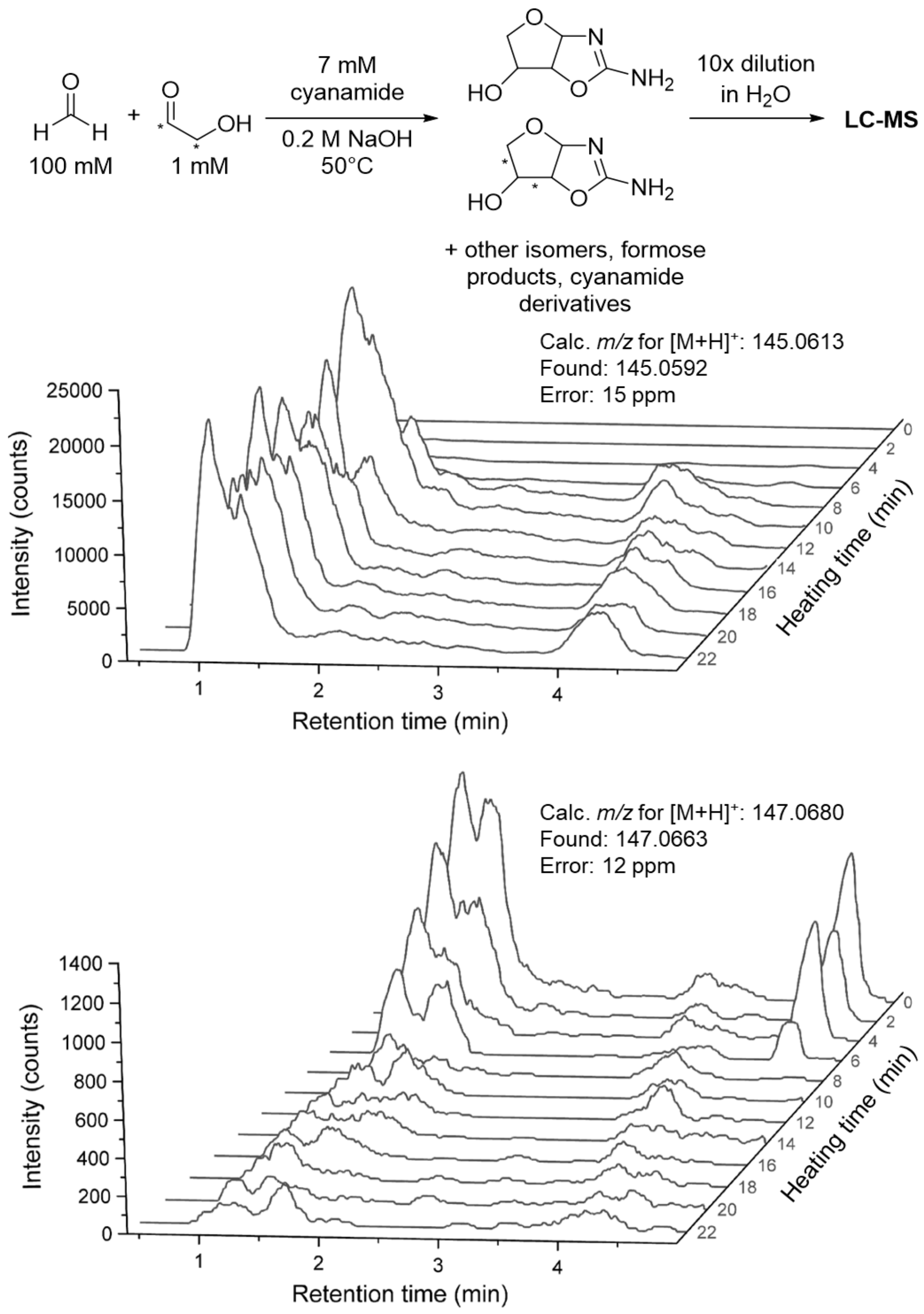

Figure S26. Extracted ion chromatograms of $C_5H_8N_2O_3$ (C_4-cyanamide adduct anhydrate) in formose reaction timepoints containing 7 mM cyanamide and 1 mM initial glycolaldehyde-1,2-$^{13}C_2$. A mass range of +/− 10 ppm, based on found mass, was applied for each EIC. The concentration of the doubly-^{13}C-labelled species (m/z_{obs} 147.0663), which formed predominantly at the start, remained relatively constant. The unlabelled species (m/z_{obs} 145.0592) started at 0 and increased significantly at the onset of the exponential phase. After 10 minutes, the concentration plateaued.

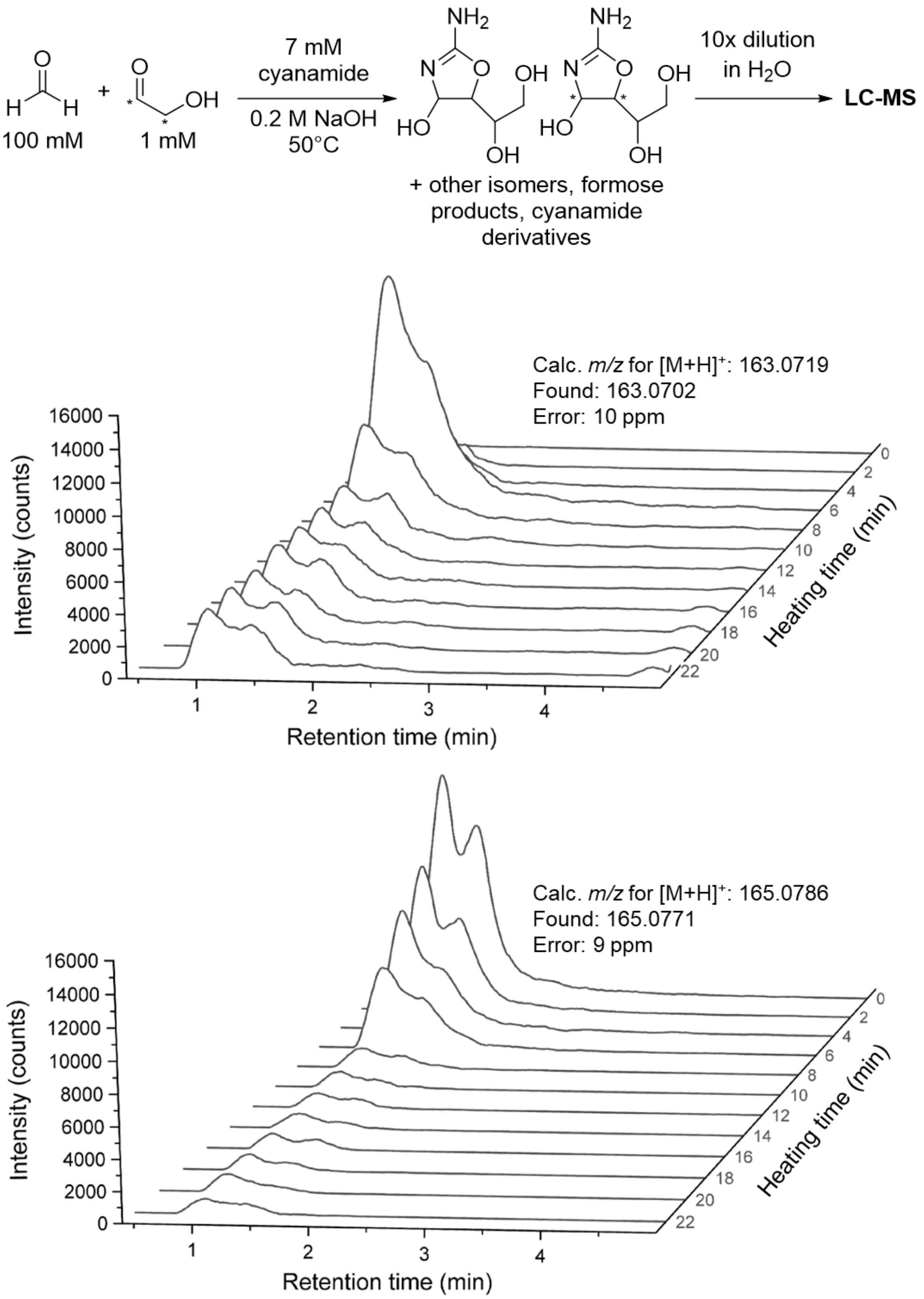

Figure S27. Extracted ion chromatograms of $C_5H_{10}N_2O_4$ (C_4-cyanamide adduct) in formose reaction timepoints containing 7 mM cyanamide and 1 mM initial glycolaldehyde-1,2-$^{13}C_2$. A mass range of +/– 10 ppm, based on found mass, was applied for each EIC. See Table S1 for proposed structures of possible aminooxazole derivatives. The concentration of the doubly-^{13}C-labelled species (m/z_{obs} 165.0771), which formed predominantly at the start, decreased continuously throughout the experiment. The unlabelled species (m/z_{obs} 163.0702) started at 0, reached maximum concentration during the exponential phase (~6 min), decreased until 10 minutes, and plateaued afterwards, which reflected change of the anhydrate.

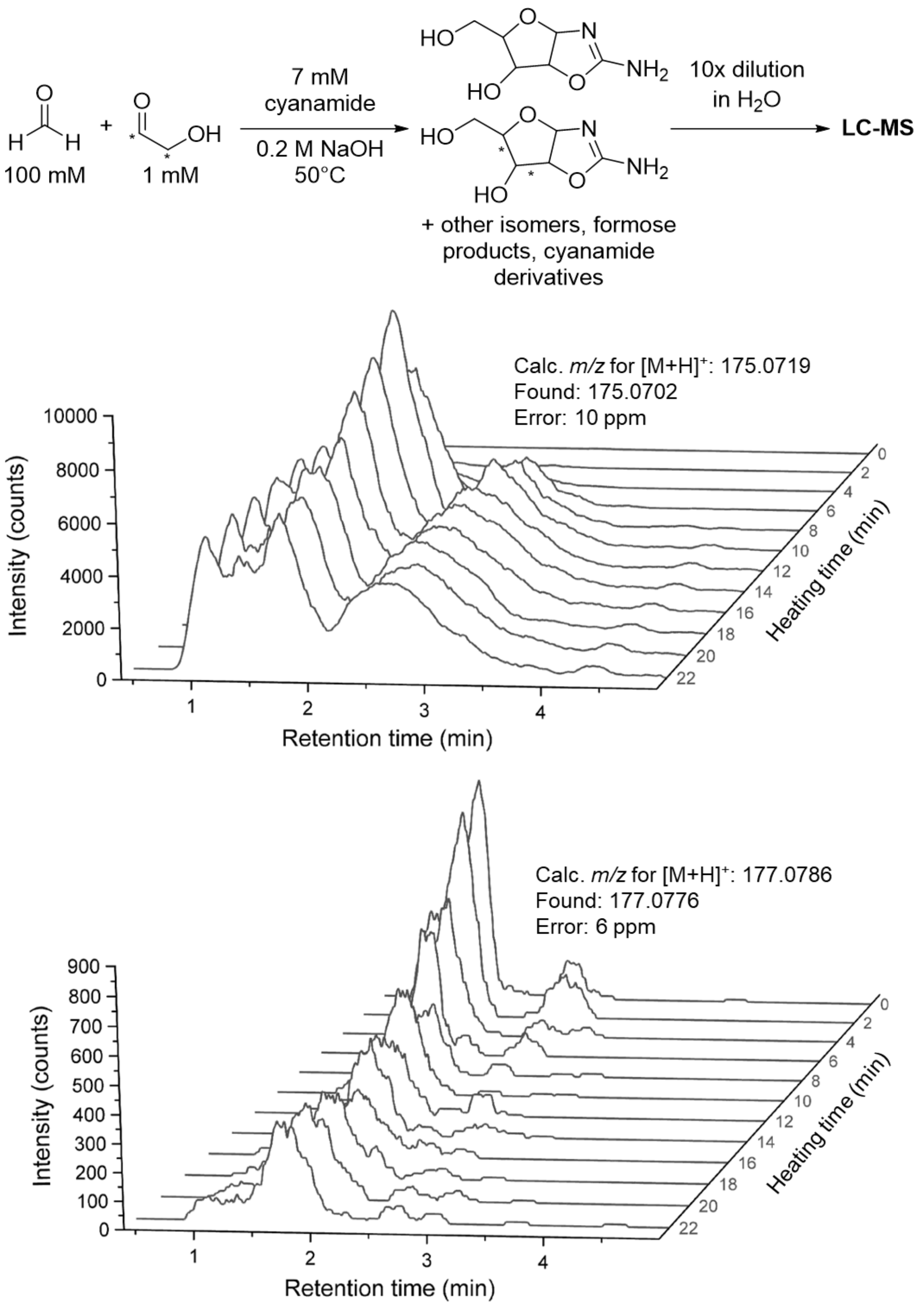

Figure S28. Extracted ion chromatograms of $C_6H_{10}N_2O_4$ (C_5-cyanamide adduct anhydrate) in formose reaction timepoints containing 7 mM cyanamide and 1 mM initial glycolaldehyde-1,2-$^{13}C_2$. A mass range of +/− 10 ppm, based on found mass, was applied for each EIC. See Table S1 for proposed structures aminooxazoles derivatives. The concentration of the doubly-^{13}C-labelled species (m/z_{obs} 177.0776), which formed predominantly at the start, remained relatively constant. The unlabelled species (m/z_{obs} 175.0702) started at 0 and increased significantly at the onset of the exponential phase. After 10 minutes, the rate of increase slowed down.

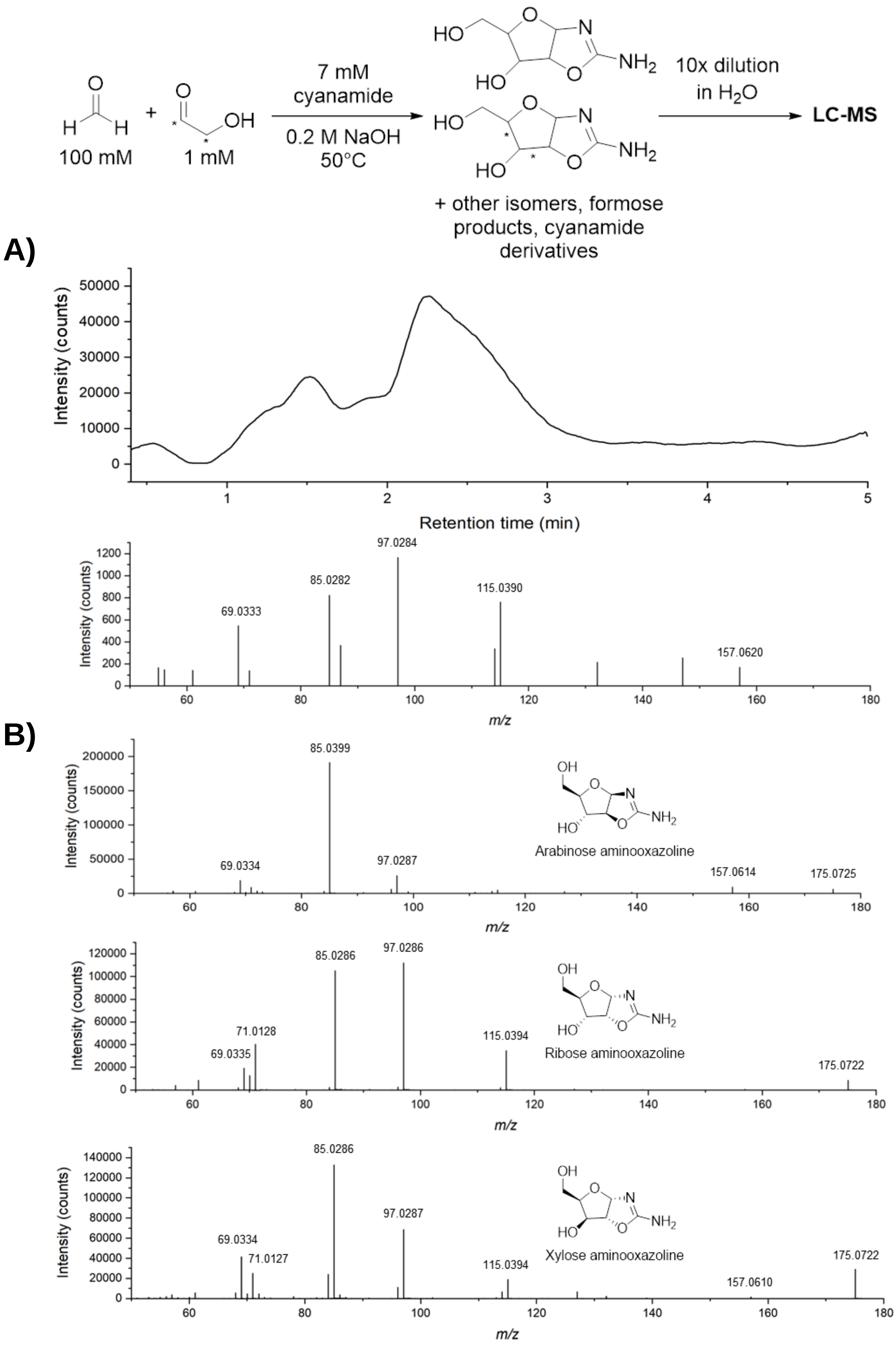

Figure S29. LC-MS/MS analysis of the formose reaction containing 7 mM cyanamide and 1 mM initial glycolaldehyde-1,2-$^{13}C_2$ at the 22-minute timepoint. **A)** Total ion chromatogram of m/z_{calc} 175.0719 MS/MS (middle) and mass spectrum at RT 1.023 min (bottom). MS/MS spectrum of m/z_{calc} 175.0719 at RT 1.023 min, showing many of the same fragmentation peaks observed in MS/MS spectra of pentose aminooxazoline standards shown in **B)**. arabino- (top), ribose- (middle), and lyxose aminooxazoline (bottom) standards shown in Supplementary Figures S6–8.

7. Time course experiment of formose reaction with 4 mM 2-aminooxazole

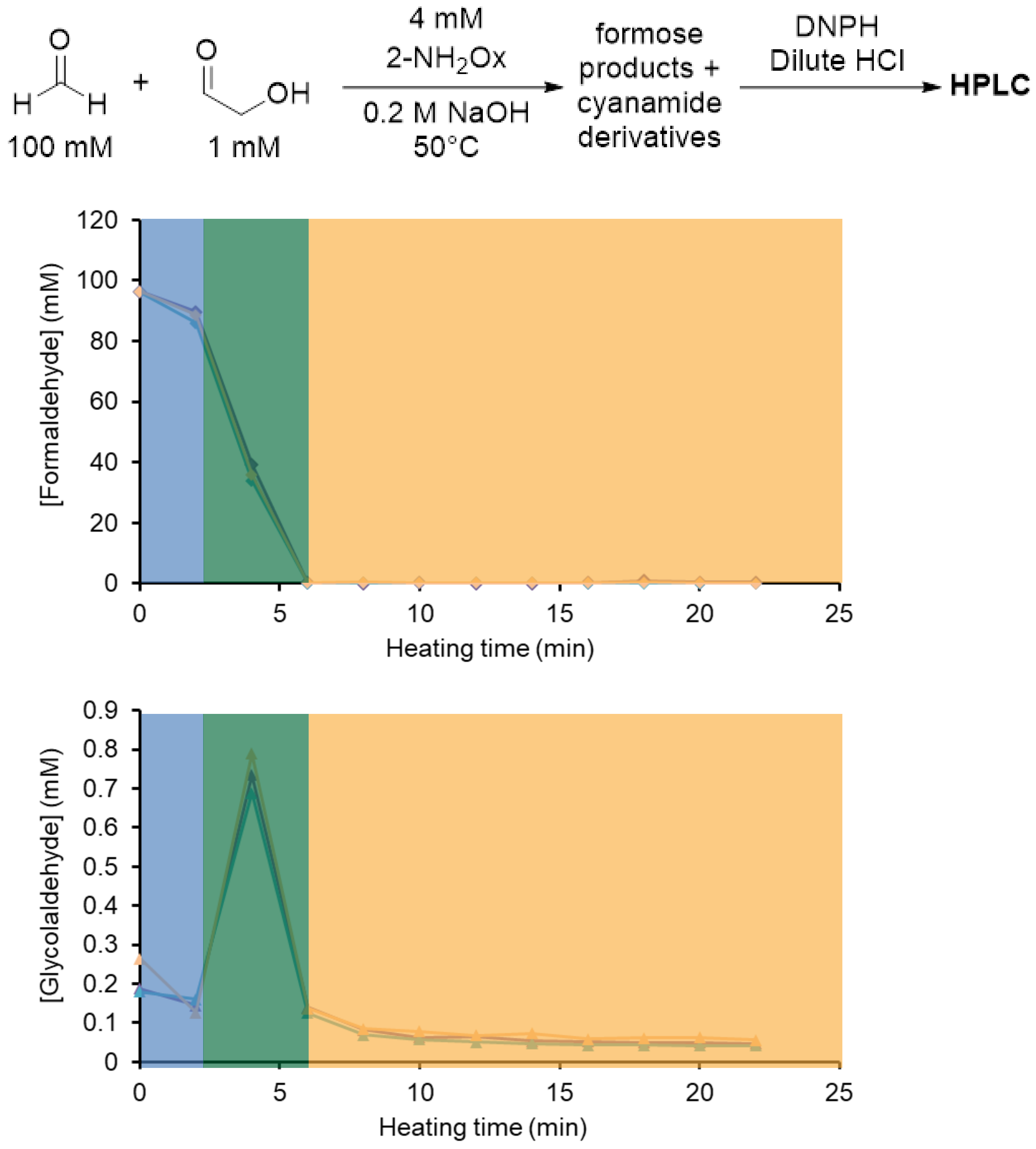

Figure S30. Concentrations of formaldehyde and glycolaldehyde over time in triplicates of the formose reaction containing initially 4 mM 2-NH_2Ox. Concentrations of formaldehyde (middle) and glycolaldehyde (bottom) were determined through derivatisation with DNPH followed by analysis with HPLC (see Figure S31 for extracted HPLC chromatograms). The regions are colour-coded based on their kinetic phase–lag phase (blue), exponential (green), and degradation (yellow). Their concentrations were calculated based on their respective standard curves (see Figure S2 and S3). The changes in [formaldehyde] and [glycolaldehyde] resemble that of the formose reaction without any cyanamide, suggesting no or a negligible effect on the kinetics.

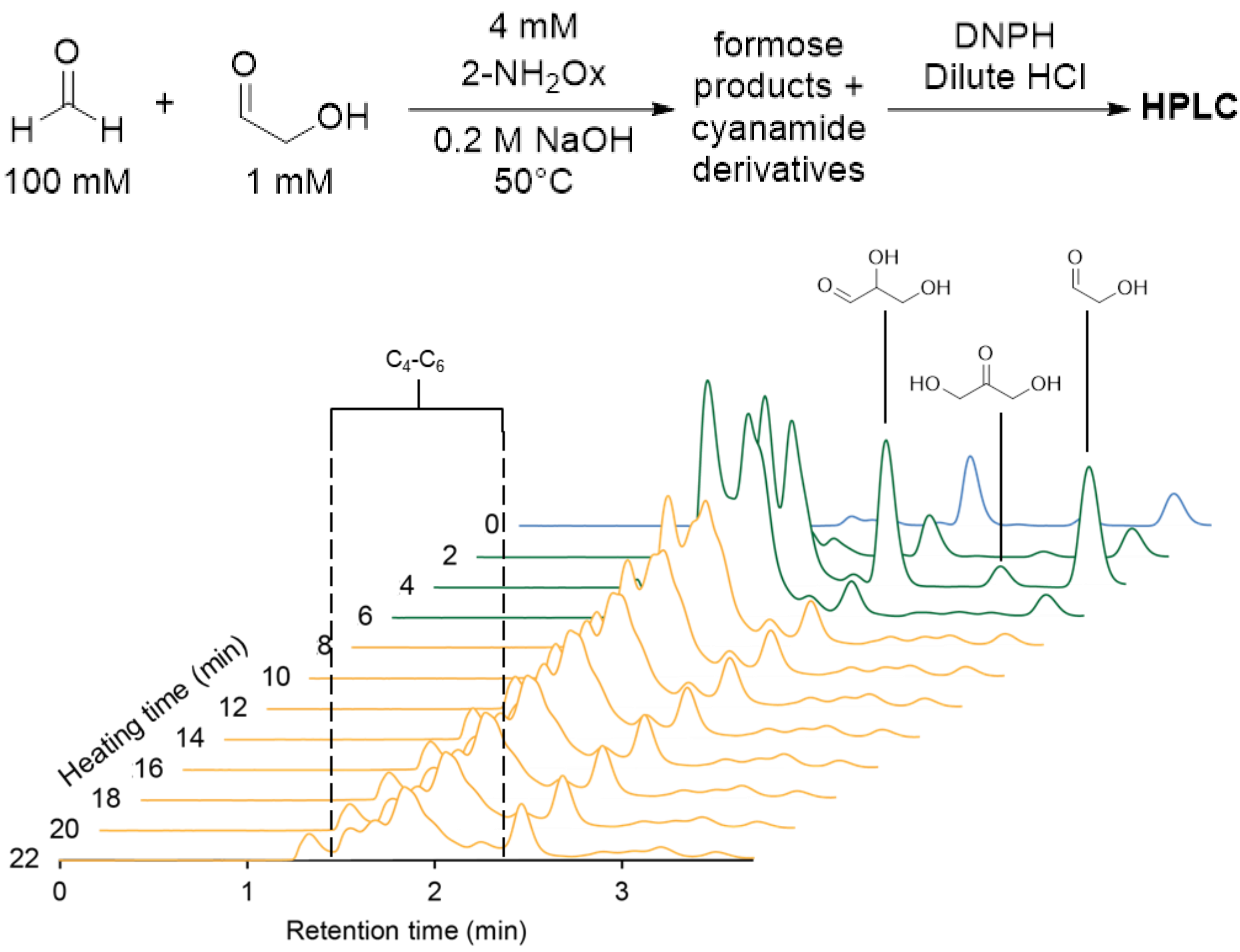

Figure S31. Extracted HPLC chromatograms at 360 nm absorbance of formose reaction timepoints containing initially 4 mM 2-aminoxoazole. The chromatograms are colour coded based on which phase of the formose reaction they belong to. Blue chromatograms represent the lag phase, green the exponential phase, and yellow the degradation phase. With 4 mM initial 2-NH_2Ox, the changes in product distribution as a function of time were similar to that of the formose reaction without any cyanamide. After the yellowing point (~6 min), the peaks gradually decrease in intensity.

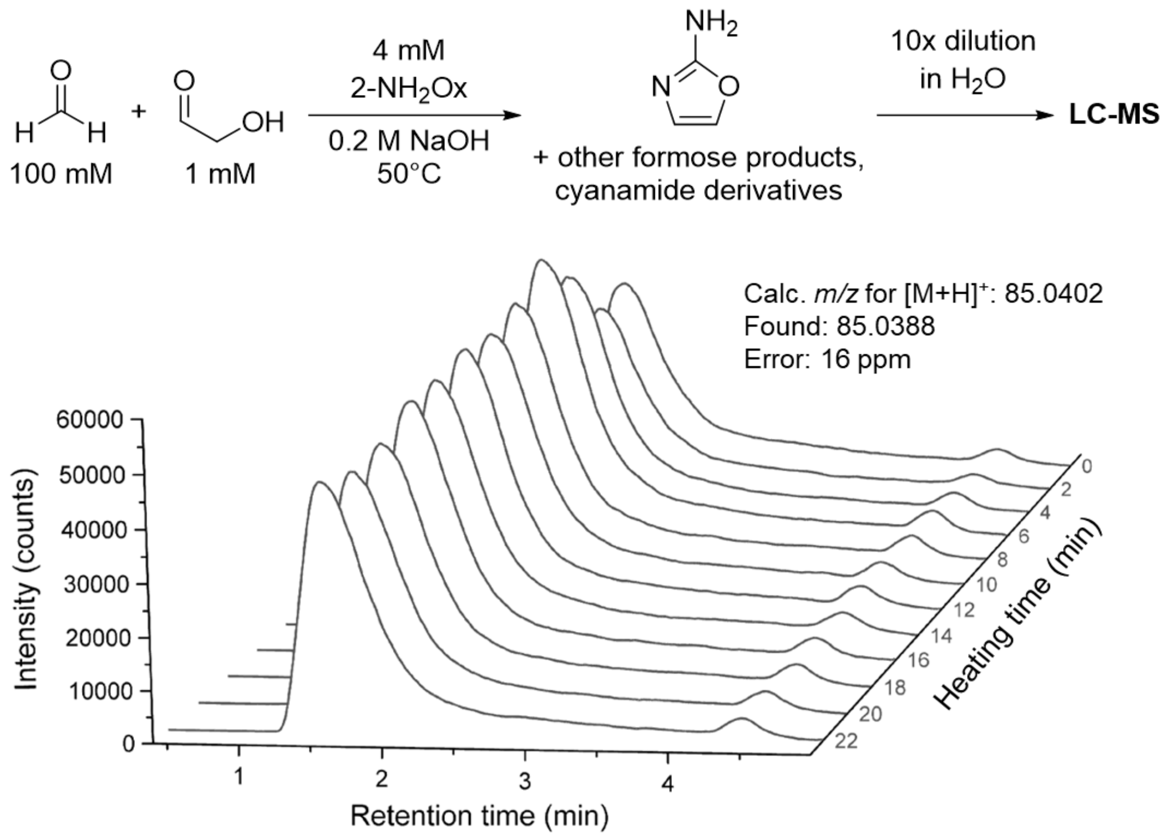

Figure S32. Extracted ion chromatograms of $C_3H_4N_2O$ (2-NH_2Ox) in formose reaction timepoints containing initially 4 mM 2-aminooxazole. A mass range of +/– 10 ppm, based on found mass, was applied to each EIC. [2-NH_2Ox] started to increase after 2 minutes of heating and plateaued at 6 minutes. It is hypothesised that the increase in [2-NH_2Ox] resulted from the breakdown of unstable adducts of 2-NH_2Ox and components of the formose reaction.

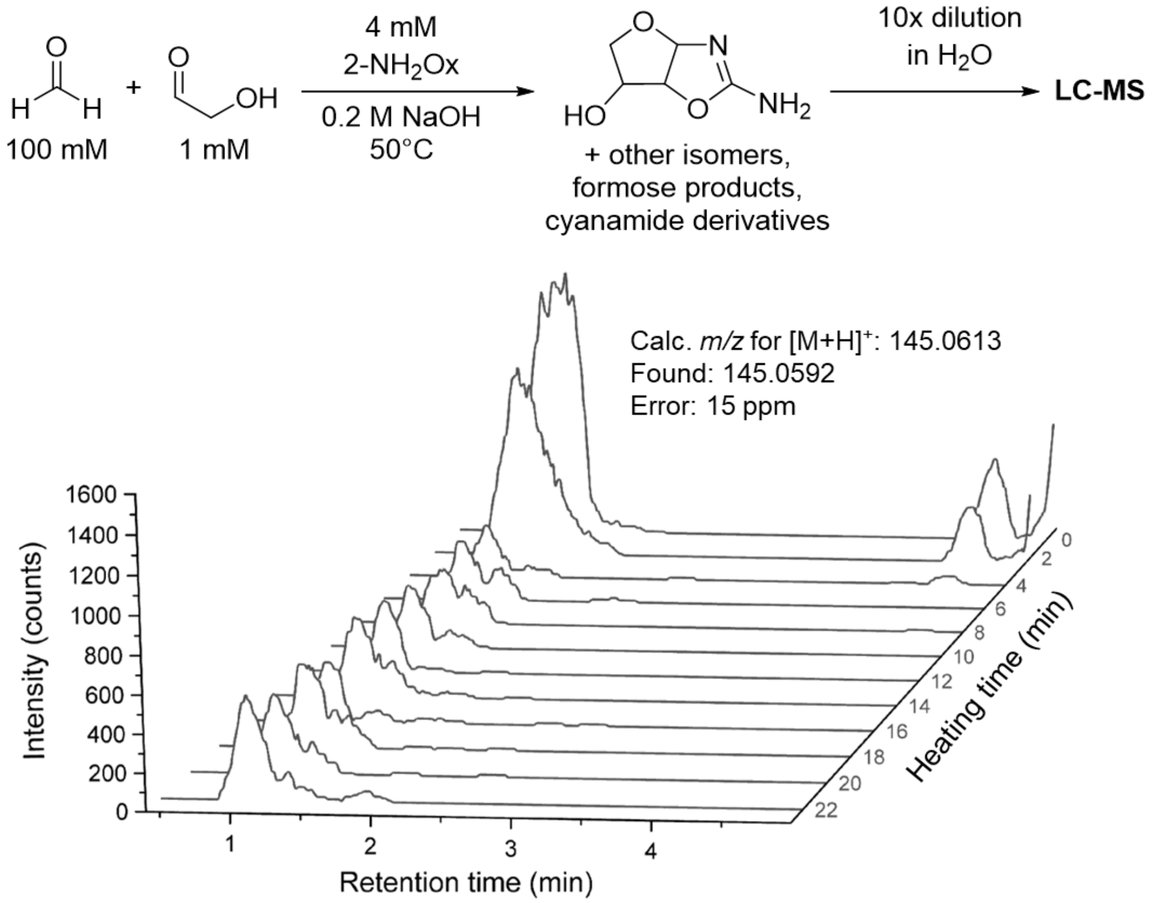

Figure S33. Extracted ion chromatograms of $C_5H_8N_2O_3$ (2-NH_2Ox-glycolaldehyde adduct) in formose reaction timepoints initially containing 4 mM 2-aminooxazole. A mass range of +/– 10 ppm, based on found mass, was applied to each EIC. The intensity of all observed peaks plummeted once the mixture was heated suggesting the adducts were unstable. Only the peak at ~ 1.1 minutes retention time was observed to gradually increase over the course of the experiment

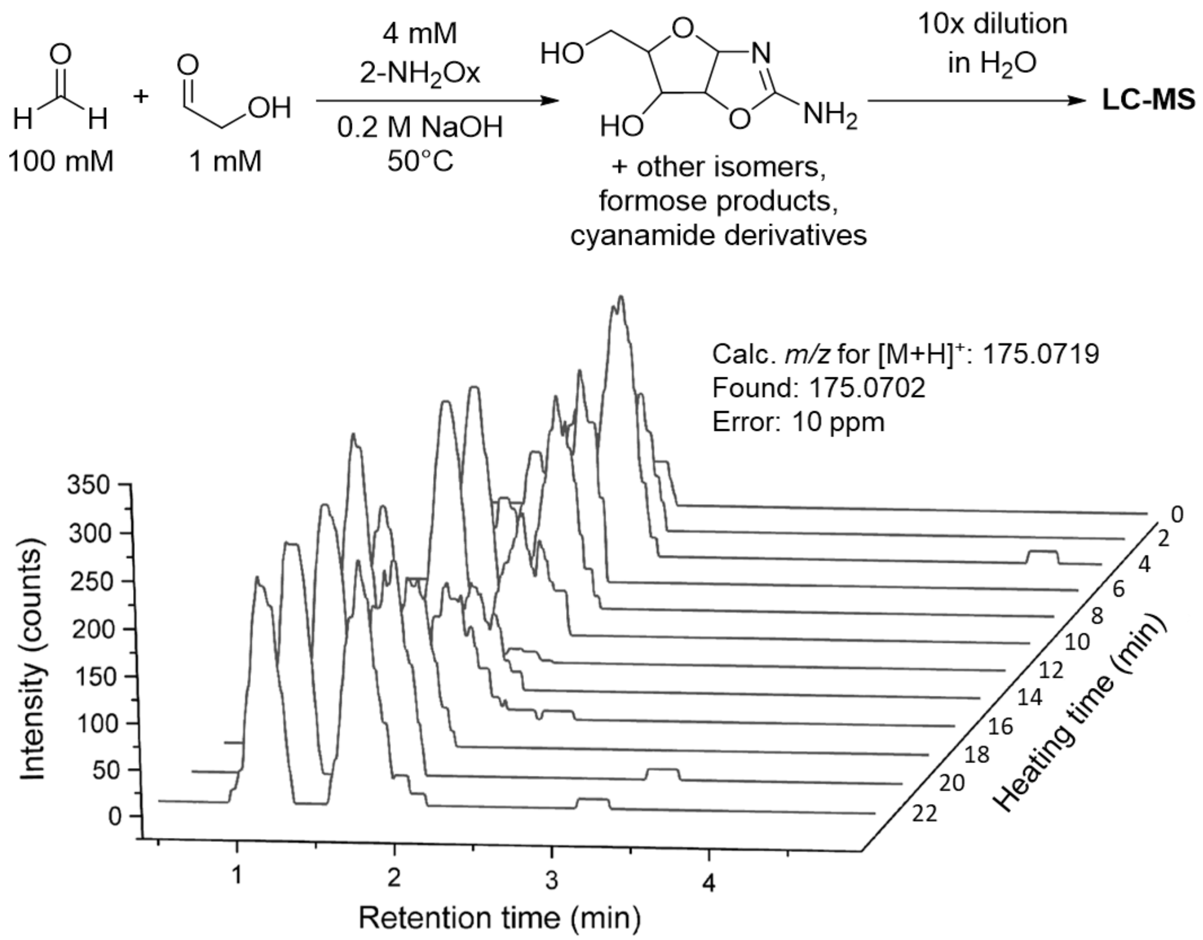

Figure S34. Extracted ion chromatograms of $C_6H_{10}N_2O_4$ (2-NH_2Ox-glyceraldehyde adduct) in formose reaction timepoints initially containing 4 mM 2-aminooxazole. A mass range of +/– 10 ppm, based on found mass, was applied to each EIC. The intensity of the peaks remained low throughout the observation window suggesting that the formation of pentose aminooxazolines via the reaction between glyceraldehyde and 2-NH_2Ox is unlikely the dominant pathway.

Table S3. Structures of aminooxazole and aminooxazoline derivatives proposed to form from cyanamide-mediated formose reaction.

Observed *m/z*	Proposed Structure	Reported?	Selected References
115.0497	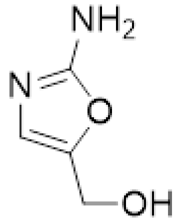	**Y**	1,2
115.0497	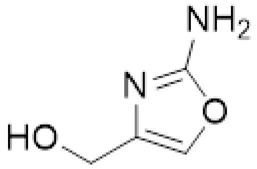	**N**	
115.0497	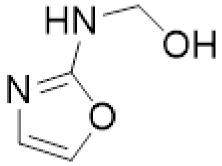	**Y**	1
133.0599	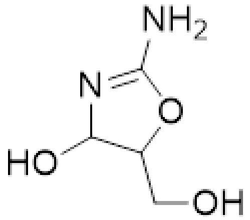	**Y**	1,2
133.0599	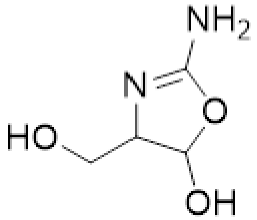	**N**	
145.0592	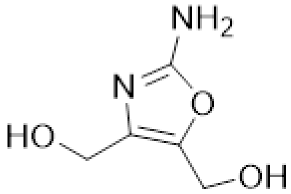	**N**	
145.0592	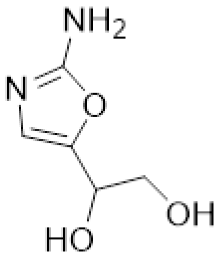	**N**	

145.0592	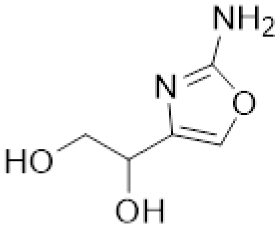	**N**	
145.0592	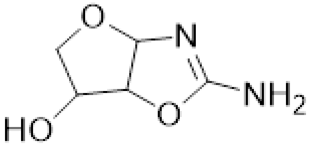	**Y**	1–5
145.0592	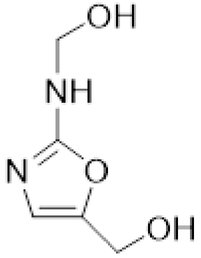	**N**	
163.0702	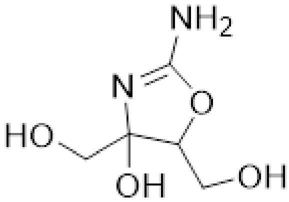	**N**	
163.0702	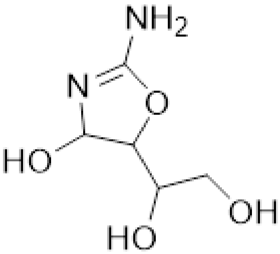	**N**	
163.0702	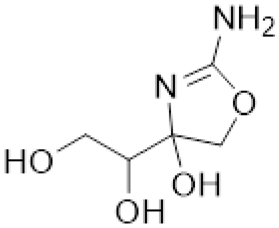	**N**	
175.0702	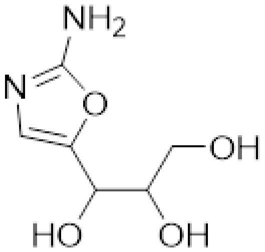	**Y**	6,7

175.0702	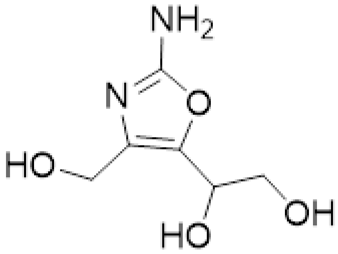	**N**	
175.0702	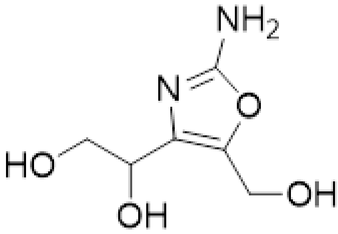	**N**	
175.0702	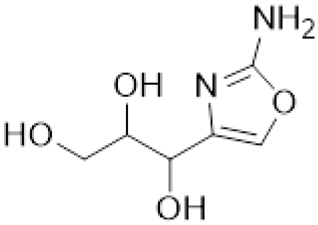	**N**	
175.0702	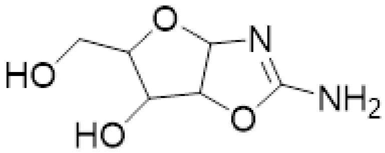	**Y**	1,3–8
175.0702	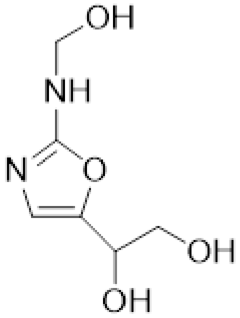	**N**	

8. Yellowing Point Assay of the Formose Reaction with 10 mM Sodium Cyanide

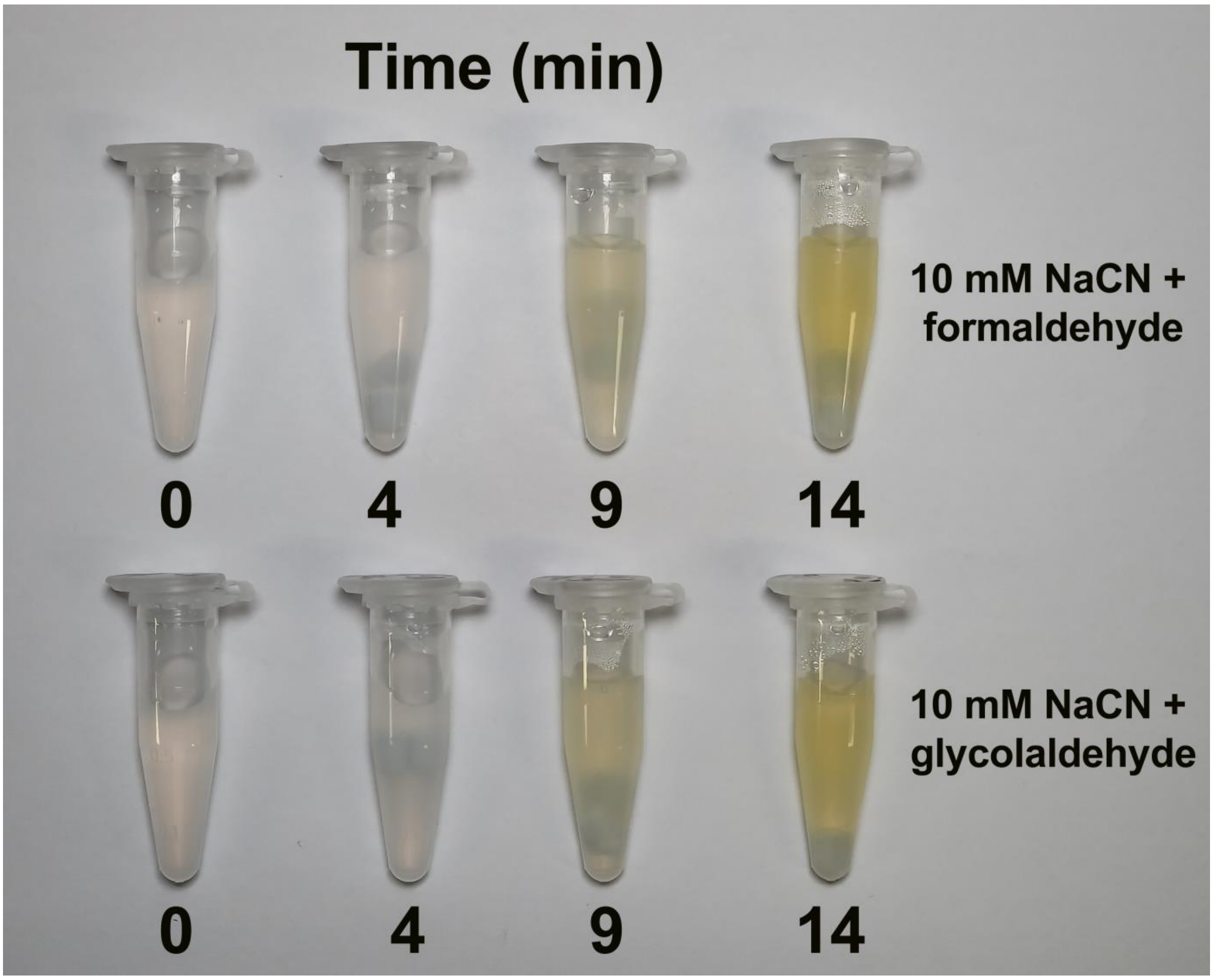

Figure S35. Time-course experiment of formose reaction mixtures containing initial 100 mM CH_2O, 1 mM glycolaldehyde, 0.2 M NaOH, 30 mM calcium acetate, and 10 mM NaCN heated to 50 °C. Sodium cyanide was mixed with CH_2O first in the first time course (top row) and glycolaldehyde first in the second (bottom row). The mixtures in both experiments reached the yellowing point ~8–9 minutes, suggesting that this concentration of sodium cyanide does not significantly affect the formose reaction kinetics.

9. Control Experiments and Discussion Regarding Hemiaminal Formation in Formose Reaction Samples

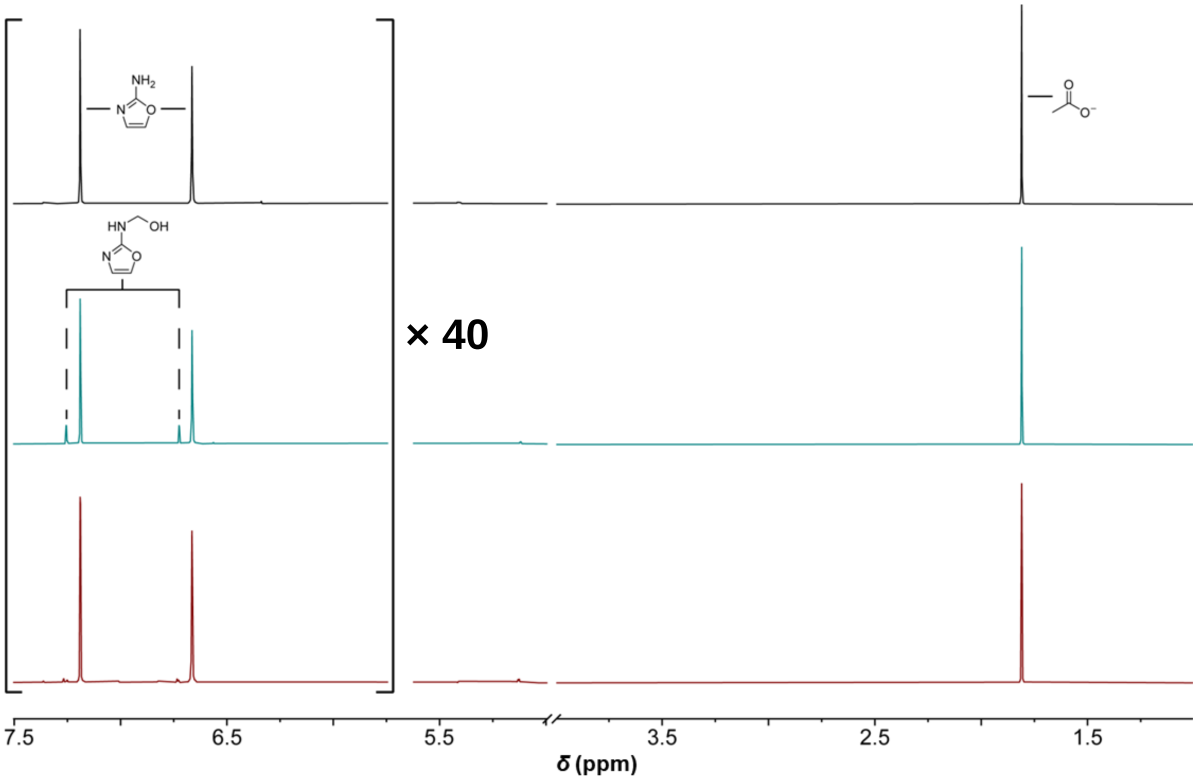

Figure S36. ^{1}H NMR spectrum of 2-NH_2Ox and its hemiaminal product with CH_2O in 0.2 M NaOH and 30 mM calcium acetate. A 1 mL mixture of 10 mM 2-NH_2Ox and 10 mM CH_2O in 0.2 M NaOH, 30 mM calcium acetate, and 10% D_2O was left to react at room temperature for 2 hours before 100 µL of the mixture was added to 900 µL of water with 10% D_2O. Both Eppendorf tubes were incubated at room temperature for another hour before analysis via ^{1}H NMR spectroscopy and LC-MS. Top) 10 mM 2-NH_2Ox standard; Middle) 10 mM 2-NH_2Ox and 10 mM CH_2O; Bottom) The 10 mM 2-NH_2Ox and 10 mM CH_2O mixture diluted 1 in 10 in 10% D_2O. The spectrum is scaled based on the acetate peak for ease of comparison of the 2-NH_2Ox and hemiaminal signals. The 5.75–7.50 ppm region is magnified 40 times with respect to the acetate peak.

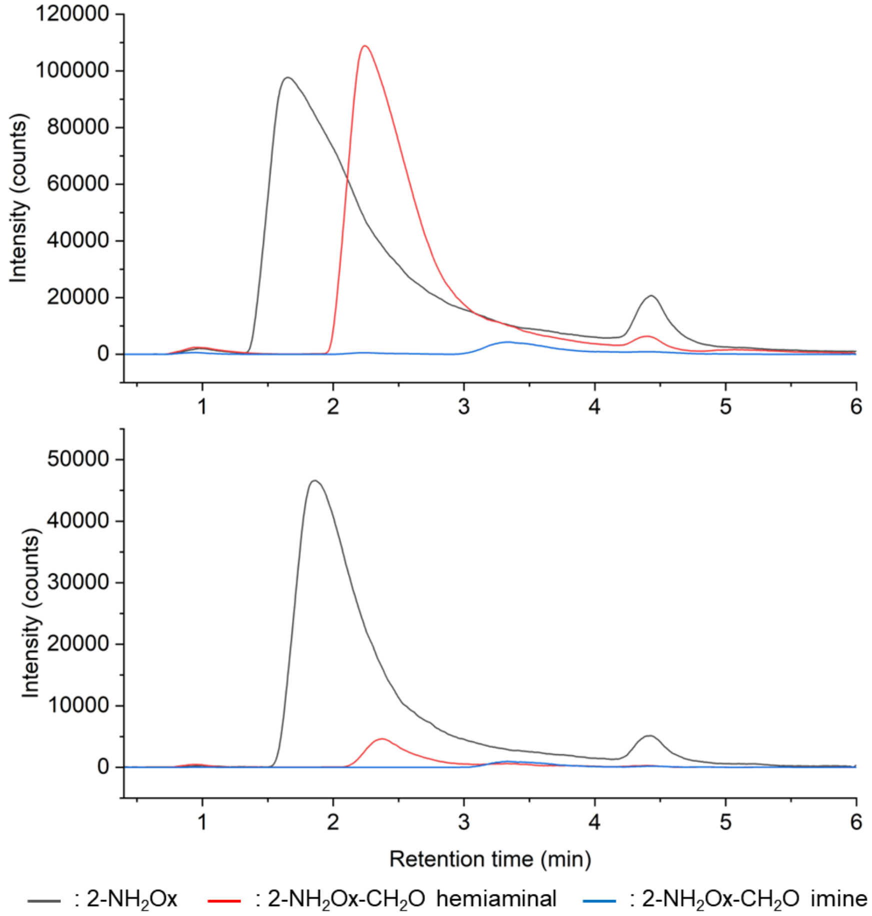

Figure S37. Extracted ion chromatograms of 2-NH_2Ox (*m/z* 85.0393 +/- 10 ppm) and its hemiaminal (*m/z* 115.0495 +/- 10 ppm) and imine (*m/z* 97.0392 +/- 10 ppm) products with formaldehyde. Top) A mixture of 10 mM 2-NH_2Ox and 10 mM CH_2O in 0.2 M NaOH and 30 mM calcium acetate diluted 1 in 100 for LC-MS analysis. Bottom) The aforementioned mixture diluted 1 in 10, allowed to equilibrate for one hour, and subsequently diluted 1 in 100 for LC-MS analysis.

Discussion. The ^{1}H NMR spectra (Figure S36) show the peaks for 2-NH_2Ox and what is likely its hemiaminal product with formaldehyde. Figure S37 shows the mass chromatograms for *m/z* values of 2-NH_2Ox and its hemiaminal and imine products with formaldehyde. Dilution is observed to shift the equilibrium towards free formaldehyde and 2-NH_2Ox. These results suggest that some fraction of formose-derived aldehydes likely exist as hemiaminals during the formose reaction, a smaller fraction of which undergoes dehydration to form their respective imines. Therefore, HPLC and LC-MS data of formose reaction mixture likely reflects higher concentrations of free aldehydes and amines than what is present during the formose reaction. However, the concentration of hemiaminal observed is relatively small, and equilibria between free aldehydes and hemiaminals are reversible. As a consequence, the HPLC and LC-MS data on formose reaction mixtures nonetheless represents the effective concentrations of free aldehydes and amines that are ultimately available in the reaction system.

References

1 M. W. Powner, J. D. Sutherland and J. W. Szostak, *J. Am. Chem. Soc.* **2010**, *132*, 16677–16688.

2 S. Islam, J. A. Aguilar, M. W. Powner, M. Nilsson, G. A. Morris and J. D. Sutherland, *Chem. Eur. J.* **2013**, *19*, 4586–4595.

3 C. Anastasi, M. A. Crowe, M. W. Powner and J. D. Sutherland, *Angew. Chem.* **2006**, *45*, 6176–6179.

4 S. Islam, D. K. Bučar and M. W. Powner, *Nat. Chem.* **2017**, *9*, 584–589.

5 B. W. F. Colville and M. W. Powner, *Angew. Chem.* **2021**, *60*, 10526–10530.

6 M. W. Powner, B. Gerland and J. D. Sutherland, *Nature*, **2009**, *459*, 239–242.

7 M. W. Powner and J. D. Sutherland, *Angew. Chem.* **2010**, *49*, 4641–4643.

8 R. A. Sanchez and L. E. Orgel, *J. Mol. Biol.* **1970**, *47*, 531–543.
